# Nationwide Monitoring and Hepatic Mixture Risk Assessment of PFASs in Korean Drinking Water Using Relative Potency Factors

**DOI:** 10.3390/toxics14070577

**Published:** 2026-06-30

**Authors:** Yubeen Kim, Shervin Hashemi, Heesoo Pyo, Youngwook Lim, Changsoo Kim, Incheol Choi, Jiyeon Yang

**Affiliations:** 1 Graduate School of Public Health, Yonsei University, 50-1 Yonsei-ro, Seodaemun-gu, Seoul 03722, Republic of Korea; kyb8542@kosha.or.kr; 2 Inhalation Toxicity Research Center, Occupational Safety and Health Research Institute, Korea Occupational Safety and Health Agency, Daejeon 34122, Republic of Korea; 3 College of Pharmacy, Dankook University, Cheonan-si 31116, Republic of Korea; 4 Institute for Environmental Research, Yonsei University College of Medicine, 50-1 Yonsei-ro, Seodaemun-gu, Seoul 03722, Republic of Korea; shervin@yuhs.ac (S.H.); envlim@yuhs.ac (Y.L.); preman@yuhs.ac (C.K.); 5 International Advanced Analysis Institute, B-339, 140, Tongil-ro, Deogyang-gu, Goyang-si 10594, Republic of Korea; phs3692@kist.re.kr; 6 Department of Preventive Medicine and Public Health, Yonsei University College of Medicine, 50-1 Yonsei-ro, Seodaemun-gu, Seoul 03722, Republic of Korea; 7 Water Use & Management Division, National Institute of Environmental Research, Hwangyong-ro 42, Seogu, Incheon 22689, Republic of Korea; cic00@korea.kr

**Keywords:** PFOA-equivalent risk, hazard quotient, hepatic toxicity, finished water, endpoint-specific assessment, nationwide monitoring

## Abstract

This study evaluated the reliability and application of relative potency factors (RPFs) for assessing hepatic mixture risks of per- and polyfluoroalkyl substances (PFASs) in Korean finished drinking water. A total of 1254 finished water samples collected from 70 drinking water treatment plants between 2018 and 2024 were analyzed for eight PFAS compounds. Hepatic RPFs proposed by the National Institute for Public Health and the Environment (RIVM) were assessed using a structured scoring system and applied to estimate PFOA-equivalent mixture risks. Hazard quotients (HQs) based on hepatic toxicity reference doses were also calculated for comparison. PFAS concentrations generally declined over time, including PFOA from 0.0032 to 0.0014 μg/L, PFOS from 0.0008 to 0.0003 μg/L, and PFHxS from 0.0072 to 0.0004 μg/L between 2018 and 2024. The RPF-based method produced higher cumulative risk estimates than the individual toxicity-based approach, suggesting that single-compound HQs may underestimate risks from co-occurring PFASs. Although total risks were generally below the non-carcinogenic threshold of 1.0, the 95th percentile PFOA-equivalent risk for PFNA exceeded the individual threshold of 0.1. These findings support endpoint-specific RPF-based assessment for PFAS mixtures in drinking water.

## 1. Introduction

Per- and polyfluoroalkyl substances (PFASs) constitute a group of over 4000 synthetic chemicals. Within this group, a particular subset, specifically, perfluoroalkyl acids that possess carboxylate or sulfonate functional groups, has emerged as a significant concern for drinking water due to their widespread contamination and environmental persistence [1]. These substances exhibit high resistance to degradation, enabling them to persist in the environment and human body for extended periods [2]. PFASs have been utilized in various products, including non-stick cookware, stain-resistant fabrics, waterproof clothing, firefighting foams, and food packaging materials [3,4,5,6].

The United Nations has incorporated clean water and sanitation as one of the Sustainable Development Goals (SDGs), specifically with Goal 6, aiming to ensure water and sanitation availability and sustainability [7,8]. This objective underscores the critical significance of access to potable water as a fundamental human right and cornerstone for public health, economic development, and environmental sustainability. In line with this, PFAS contamination in drinking water has emerged as a pressing issue, particularly in residential areas proximal to military installations, agricultural lands, and industrial sites where these chemicals have been extensively utilized [9,10].

One caveat in addressing PFAS contamination in drinking water is the difficulty of removing the chemicals. Conventional water treatment methods, such as chlorination or filtration, are ineffective in eliminating PFASs [11]. On the other hand, more advanced techniques, including activated carbon filtration, reverse osmosis, and ion exchange, have demonstrated efficacy in removing PFASs from drinking water; however, these methods are often cost-prohibitive and may not be accessible in all communities [12]. Furthermore, certain treatment methods may leave residual amounts of PFASs, raising concerns about the thoroughness of the remediation [13]. Another complication is the lack of an immediate perception of contamination. PFASs do not alter the organoleptic properties of water, implying that individuals may consume contaminated water obliviously [14]. Consequently, numerous municipal water systems are now implementing PFAS contamination testing, and private well owners in affected areas are advised to assess their water quality.

The persistence of PFASs in water suggests that even minimal quantities can accumulate over time, rendering it a major public health concern. The health risks associated with chronic exposure have become the primary concern regarding PFASs in drinking water. These chemicals have been associated with various health issues, including cancer (particularly kidney and testicular cancer), liver damage, immune system suppression, and hormonal imbalances [15,16]. Among these, hepatic toxicity is recognized as a significant consequence of chronic exposure to PFASs.

Due to the associated health risks, the regulation of PFASs in drinking water has emerged as a significant public health concern. On 10 April 2024, the United States Environmental Protection Agency (US-EPA) finalized regulations for six PFAS compounds after reviewing over 120,000 public comments [17]. Subsequently, on 14 May 2025, the US-EPA announced its decision to uphold the existing National Primary Drinking Water Regulations for PFOA and PFOS, extend compliance deadlines, and establish a federal exemption framework [17]. These regulations set Maximum Contaminant Levels for PFOA, PFOS, PFHxS, PFNA, and HFPO-DA (GenX Chemicals) [17].

In the Republic of Korea, access to potable water is generally characterized by high quality and extensive availability. The nation has invested in water infrastructure to ensure that most urban and rural populations can access clean and safe drinking water [18]. Accordingly, Korea has implemented measures to regulate PFASs in drinking water, although a regulatory framework remains to be developed. The Korean government, specifically the Ministry of Environment (MOE), has promoted an increased awareness of the potential hazards associated with these substances. While the country has not yet implemented comprehensive regulations on PFASs, it has established standards for monitoring specific PFAS compounds, including PFOA, PFOS, and PFHxS, in drinking water since 2018 [19]. These standards set monitoring maximum allowable limits for PFOS and PFOA at 0.07 μg/L (individually and in summation). The maximum permissible monitoring level for PFHxS is 0.48 μg/L. Thus, all Korean public water systems are strictly required to conduct PFAS contamination testing once every three months. The Republic of Korea has also intensified efforts to monitor and analyze water sources for PFAS contamination. Following the PFAS leakage incident in 2018, nationwide surveys and studies targeting public water systems and private wells were initiated to assess the PFAS levels in Korea [20,21,22]. This data collection constitutes part of a broader effort to understand the extent of PFAS contamination on a national scale.

Concurrently, health-based guidance values for PFASs have evolved, increasingly incorporating human epidemiological evidence. These values are often more stringent than earlier animal-based values and may be derived from various critical effects, including immune response, developmental outcomes, serum lipids, cancer endpoints, and liver biomarkers. For instance, EFSA established a group tolerable weekly intake for PFOA, PFOS, PFNA, and PFHxS, primarily based on reduced antibody response to vaccination [23]. In contrast, recent US-EPA toxicity assessments for PFOA and PFOS considered multiple non-cancer and cancer endpoints, including immune, developmental, cardiovascular, hepatic, and cancer-related effects [24,25]. Consequently, while these values are crucial for regulatory screening, combining or comparing values derived from different endpoints may complicate endpoint-specific mixture risk assessment.

Per- and polyfluoroalkyl substances (PFASs) are typically present in drinking water as a mixture of various compounds [26,27]. However, current hepatic toxicology data and studies are limited to a small subset of PFASs [27]. Given that PFAS compounds share similar mechanisms of liver toxicity, the relative potency factor (RPF) approach offers an effective means to evaluate the overall hepatic toxicity of PFAS mixtures commonly found in drinking water [27]. This method facilitates PFAS mixture risk assessment by converting measured concentrations of individual PFASs into PFOA-equivalent concentrations, allowing the combined effects of different compounds to be expressed as a single mixture metric [27].

The National Institute for Public Health and the Environment (RIVM) in the Netherlands has recently examined this approach [27,28]. However, there is a paucity of studies that have attempted to compare the risk assessment methodologies utilizing RIVM’s Relative Potency Factors (RPFs) with those calculating hazard quotients (HQs), despite both methods being based on similar liver toxicity parameters. Consequently, it is important to evaluate the reliability of RPF-based models for assessing liver-related risks of PFAS mixtures in drinking water. Given that RPFs are endpoint-specific, their application necessitates consistency between the selected toxicity endpoint, the index compound, and the toxicity values used for comparison.

This study had three primary objectives. First, a comprehensive long-term nationwide analysis of PFASs in Korean drinking water was conducted. Second, RPFs were evaluated using a structured scoring system and applied to the PFAS monitoring results. Third, ingestion exposure and risk assessments were performed using both the RPF-based PFOA-equivalent approach and the conventional HQ approach based on individual-substance RfDs for liver toxicity.

## 2. Materials and Methods

### 2.1. Relative Potency Factors for Hepatic Toxicity of PFASs

The RIVM has recently concentrated on evaluating the risks associated with PFAS mixture exposure in drinking water and food. Bil et al. [27] employed the RPF methodology to assess the hepatic effects of 22 PFAS compounds. The RPFs were determined by comparing the hepatic effects in male rats, which were orally exposed for 42–90 days, with those of perfluorooctanoic acid (PFOA), the index compound. The analysis encompassed various PFAS categories, including sulfonic acids, carboxylic acids, ether carboxylic acids, and fluorotelomeric alcohols.

The derived RPFs enabled the calculation of the equivalent PFOA concentration in the mixture for potential health risk assessment. This approach allows for the comparison of total PFAS exposure in terms of PFOA equivalents with health guidelines, such as tolerable daily intake or reference dose. In light of daily PFAS exposure, Bil et al. [27] emphasized the importance of evaluating combined PFAS risks.

The RPFs proposed by Bil et al. [27] were derived from hepatic effects using PFOA as the reference compound. Therefore, this study focused on PFASs for which hepatic RPFs and compatible toxicity data were available, allowing the RPF-based and HQ-based approaches to be compared within a consistent liver-toxicity framework. This study considered eight PFASs, comprising six carboxylic acids and two sulfonic acids, as target pollutants. The RPF values for each target substance, as proposed by Bil et al. [27], are listed in Table 1.

### 2.2. Finished Water Sample Collection

**Figure 1:**
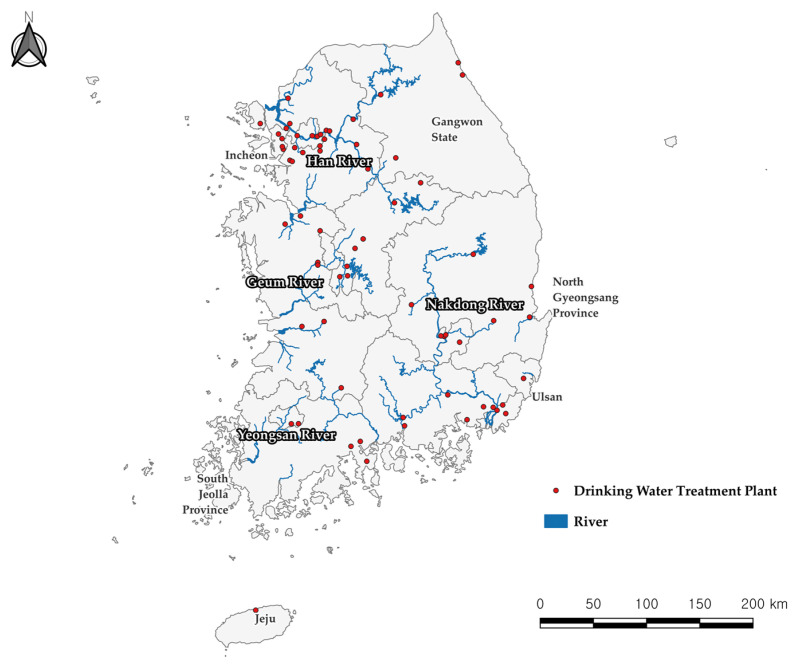
Drinking water treatment plants selected for obtaining finished water samples.

The Korea National Institute of Environmental Research (NIER) commenced an extensive monitoring program for PFASs in 2012. As depicted in Figure 1, this program includes 70 major drinking water treatment plants (DWTPs) located throughout the nation’s river basins, each with a distribution capacity exceeding 5000 m^3^/day [18]. These DWTPs consist of 58 facilities managed by local governments and 12 operated by the Korea Water Resources Corporation (K-water). They employ a variety of conventional and advanced treatment processes, such as coagulation, chlorination, filtration, ozonation, granular activated carbon treatment, UV-based advanced oxidation, and membrane filtration.

From 2018 to 2024, finished-water samples were collected from treated water at each DWTP during national monitoring campaigns, with up to three rounds per year. In each round, one sample was collected per DWTP and analyzed for all target PFAS compounds. Campaigns were distributed across spring/early summer, summer, and late summer/autumn, although exact months varied by year and DWTP depending on field logistics and monitoring schedules. Annual sample numbers included in the analysis are provided in Table S5. Samples were collected using a single-use 1 L polypropylene (PP) bottle. During sampling, 5 g of Trizma was introduced into a 1 L PP bottle, filled to maximum capacity. Samples were preserved at 4 °C and stored in a dark, cool environment devoid of organic solvent contamination. Analysis was conducted within 30 days of collection to ensure result accuracy and integrity.

### 2.3. Quantitative Determination of PFASs

The method employed solid-phase extraction (SPE) to isolate trace PFASs from water samples. Qualitative and quantitative analyses were conducted using ultra-performance liquid chromatography coupled with tandem mass spectrometry. This methodology enables accurate detection and quantification of minute PFAS concentrations in water samples. Each measurement was performed in triplicate, and arithmetic means calculated for final results.

Purified water for ultra-performance liquid chromatograph (UPLC) analysis was sourced from J.T. Baker (Center Valley, PA, USA) or purified using a Millipore Milli-Q system (Merck Millipore, Burlington, MA, USA). Only purified water, free from impurity peaks near the target compounds, was used during the blank tests to prevent contamination. UPLC-grade methanol was obtained from J.T. Baker (Center Valley, PA, USA), and precautions were taken to ensure no impurity peaks appeared near the analyte peaks during blank tests. Ammonium acetate (≥98%), used as a buffer, was purchased from Sigma-Aldrich (St. Louis, MO, USA).

Standard PFAS solutions were purchased from Wellington Laboratories (Guelph, ON, Canada) as PFAC-MXC (2000 ng/mL in MeOH). These solutions were diluted to final concentrations of 200 μg/L and 20 μg/L in methanol and stored at −20 °C. For internal standardization, individual stock solutions of MPFOA, MPFOS, MPFNA, MPFDA, MPFHxS, and MPFHxA (each 50.0 μg/mL in methanol) were also purchased from Wellington Laboratories (Guelph, ON, Canada) and mixed to prepare an internal standard solution at a concentration of 400 μg/L, which was stored at −20 °C. During the analysis, 20 μL of this internal standard mix was spiked into each 200 mL sample.

Solid-phase extraction was performed using Oasis HLB cartridges (0.2 g) purchased from Waters (Milford, MA, USA), and a 0.2 μm membrane filter (Adventec, Dundas, ON, Canada) was used to filter the final extracts. The solid-phase extraction procedure was performed using a Visiprep™ SPE Vacuum Manifold (Supelco, Bellefonte, PA, USA) and an A-100S Vacuum Pump (Eyela, Rikakikai, Tokyo, Japan). A vortex mixer (VM-10, Daihan Scientific, Wonju-si, Republic of Korea) was utilized for sample homogenization, and nitrogen evaporation was conducted using an XcelVap Nitrogen Evaporator (Horizon Technology, CA, USA). Liquid chromatographic analysis was carried out on an UltiMate 3000 UPLC system (Thermo, Waltham, MA, USA) coupled to a TSQ Quantis tandem mass spectrometer (MS/MS) from Thermo (Waltham, MA, USA). Data were analyzed using Trace Finder 4.1 General Quan software.

The sample pre-treatment procedure, depicted in Figure S1, commences with the transfer of 200 mL of the water sample into a polypropylene container. A 20 μL aliquot of the internal standard solution (400 μg/L) was then spiked into the sample. The HLB cartridges were conditioned by passing 5 mL of methanol and 6 mL of reverse osmosis water through the cartridges. After conditioning, the 200 mL sample was passed through the HLB cartridge at a flow rate of approximately 13 mL/min, and the cartridge was vacuum-dried for 10 min to remove excess liquid.

Subsequently, 5 mL of methanol was utilized to elute the PFAS from the cartridge, and the eluate was collected in a 15 mL polypropylene conical tube. The eluted solution was concentrated to dryness under a nitrogen atmosphere at 50–65 °C. Upon desiccation, the residue was reconstituted with methanol (0.5 mL) to facilitate analyte dissolution. The reconstituted sample was then filtered through a 0.2 μm membrane filter, and the resultant filtrate was transferred to a polypropylene autosampler vial for analysis via UPLC-MS/MS.

Then, 5 μL of the prepared sample was injected into a liquid chromatograph for the analytical procedure. The concentrations of the individual PFASs were determined by comparing the peak areas from the chromatogram to the calibration curve. Analysis was conducted using a UPLC-MS/MS system. Chromatographic separation was achieved using Hypersil GOLD™ C18 Selectivity LC columns (2.1 mm × 100 mm, 1.9 μm particle size, Thermo Fisher Scientific Inc., Waltham, MA, USA). The oven temperature was maintained at 40 °C, and the mobile phase comprised 5 mM ammonium acetate in water (Buffer A) and methanol (Buffer B). The flow rate was set to 0.3 mL/min (constant flow), and the injection volume was 5 μL.

Standard calibration solutions were prepared from a PFAC-MXC stock solution (2000 ng/mL in methanol) obtained from Wellington Laboratories (Guelph, ON, Canada). These solutions were diluted to 200 μg/L and 20 μg/L concentrations to plot the calibration curve and were used as working standards. The calibration standards were prepared by spiking known volumes of the standard solution into 200 mL of blank water, achieving concentrations ranging from 0.5 ng/L to 100 ng/L, with higher concentration samples reaching up to 400 ng/L. After pre-treatment, the samples were analyzed using UPLC-MS/MS, establishing the relationship between compound concentration and chromatographic peak area that provides the basis for quantifying PFASs in the water samples. The details on quality assurance and quality control for the measurement method are presented in the Supplementary Materials.

### 2.4. Reliability Evaluation of Relative Potency Factors

A reliability analysis of the toxicity data was conducted by referring to the confidence scoring system for non-cancer elements presented by Beck et al. [29] to demonstrate the rationale for selecting toxicity values based on the RPF approach for the eight target PFASs. As detailed in Table S6, the assessment process ranges from 4.4 to 25 points and is divided into two main categories: data reliability (2–10 points) and study confidence (2.4–15 points).

The data reliability category encompassed peer review, point of departure (POD) assessment, and read-across applications. In order to quantitatively evaluate the reliability and transparency of the data, a specific score of 5 points was allocated to toxicity values that are verifiable through peer-reviewed experiments. When POD was presented through a benchmark dose (BMD) approach, higher scores were assigned when the difference between the BMD value and its lower confidence limit (BMDL) was less than two-fold. The analyzed concentration distribution was used to assess the read-across applications. The PFOA equivalent concentrations for the lower and upper limits of the RPF values and their geometric means were calculated for each measurement. Then, the equivalent PFOA concentration range utilizing the lower and upper limits of the RPF values was compared with the range of equivalent PFOA concentrations using the geometric mean ±1 SD, ±2 SD, and ±3 SD of the corresponding PFOA equivalent concentration distribution. Higher scores were assigned when the equivalent PFOA concentration range utilizing the lower and upper limits of the RPF values fell within the range of equivalent PFOA concentrations using the geometric mean ±1 SD of the corresponding PFOA equivalent concentration distribution.

Regarding the reliability of the animal study data, factors such as compliance with good laboratory practice (GLP), appropriateness of toxicity testing (i.e., test species, exposure duration, administration method, statistical significance, number of animals and sex, test type, and dose setting pre-tests), and general information about the substance were considered. These elements are also integral to the basic requirements for assessing the reliability of toxicity test data submitted under the Korean chemical registration laws [30].

GLP refers to a series of processes that ensure the reliability of the toxicity testing process and results. Higher scores were allocated to studies that complied with GLP. In terms of the appropriateness of toxicity testing, scores were assigned based on the species utilized in the studies, with human epidemiological data, nonhuman primates, and mice/rats receiving the highest and lowest scores, respectively. Studies considering the half-life of PFASs in determining exposure duration had higher scores, particularly if chronic toxicity data were provided. The administration method and dose were also significant factors, as the route of administration could influence the absorption of the test substance. For instance, high doses (e.g., 40 mL/kg) may overload the gastrointestinal system and cause rapid movement through the small intestine or backflow through the esophagus [31]. Thus, oral administration via a dosing tube is preferred to ensure accurate dosing and minimize the risk of accidents [32]. Furthermore, the solvents utilized in animal studies were evaluated to optimize the internal exposure to the test substance without altering its biological or physical characteristics, and they should not exhibit toxicity to the animals [32,33]. Consequently, the optimal solvent was biologically inert and did not affect the properties of the test substance.

The number of animals and sex were evaluated, emphasizing their statistical significance, with higher scores assigned to studies involving at least 10 animals per group, including both male and female subjects. The test type was another significant factor, with higher scores allocated to repeated-dose toxicity studies and those that incorporated multigenerational reproductive or developmental toxicity research. Pre-tests for dose setting received higher scores, particularly for novel substances, considering scientific and ethical considerations. Utilizing in vitro tests and a limited number of animals for pre-testing was also recommended [32].

### 2.5. PFAS Exposure and Risk Assessment

The exposure and risk assessment conducted in this study was structured as an endpoint-specific methodological comparison, rather than as a derivation of new regulatory values or an identification of the most conservative health-based value for each PFAS. Hepatic toxicity was chosen as the common endpoint because the RPFs utilized in this study were derived from liver-effect data using PFOA as the index compound [27]. Consequently, individual-substance hazard quotients and RPF-based PFOA-equivalent risks were compared within a consistent hepatic toxicity framework. This design aimed to avoid the combination of toxicity values based on different points of departure or critical effects, which could obscure the interpretation of the RPF approach.

#### 2.5.1. Exposure and Risk Assessment Using Hazard Index Approach

Under the hazard index (HI) approach, the average daily dose (ADD, μg/kg/day) was calculated using Equation (1) to evaluate ingestion exposure to the target PFAS [18,34,35].

(1) $$ADD=\frac{C\times IR\times EF\times ED}{AT}$$

In Equation (1), C (μg/L) represents the mass concentration of PFASs in finished water, IR (L/day/kg-BW) denotes the daily water ingestion rate per unit of body weight, ED (=1 year) signifies the exposure duration, EF (=365 day/year) indicates the exposure frequency and AT (=365 days) represents the average time of exposure. Based on the Korea National Health and Nutrition Examination Surveys (KNHANES) conducted from 2013 to 2023, the IR is considered 0.016 L/day/kg-BW [18,35,36,37]. In instances where C was not detected, half of the method detection limit (MDL) was used as the PFAS concentration.

The non-carcinogenic risk of exposure to PFASs was calculated using Equation (2), where HQ represents the hazard quotient, and RfD (μg/kg/day) refers to the reference dose of the target substance [34,35].

(2) $$HQ=\frac{ADD}{RfD}$$

Table 2 shows the reference dose (RfD) values for each PFAS concerning hepatic toxicity. The RfDs used in the hazard quotient (HQ) comparison align with hepatic toxicity endpoints to ensure consistency with the liver-effect basis of the relative potency factors (RPFs), facilitating a coherent comparison between individual-substance HQs and PFOA-equivalent risks. This analysis is not a comprehensive regulatory risk assessment using the most stringent health-based guidance for each PFAS but an endpoint-matched evaluation of two mixture-assessment approaches. Using RfDs based on diverse critical effects would yield a more conservative screening estimate in some cases but wouldn’t directly assess hepatic RPF performance.

The Hazard Index (HI) for the total PFAS has been determined using Equation (3), where HQ_i_ represents the hazard quotient for the i-th PFAS.

(3) $$HI=\sum_{i=1}^{n}{HQ}_{i}$$

#### 2.5.2. Exposure and Risk Assessment Using Relative Potency Factor Method

Within the framework of the RIVM’s RPF method, as described by Bil et al. [27], PFOA is utilized as the reference chemical. Subsequently, the measured concentrations of each PFAS were converted to PFOA equivalents using Equation (4).

(4) $${PEQ}_{i}=C_{i}\times {RPF}_{i}$$

In Equation (4), PEQ_i_ (μg/L) denotes the PFOA equivalent concentration for the specified PFAS, C_i_ (μg/L) signifies the mass concentration of the PFAS in the treated water, and RPF_i_ represents the corresponding relative potency factor of the PFAS.

Consequently, the equivalent average daily dose for each PFAS (ADD_PEQ_) can be determined using Equation (5).

(5) $${ADD}_{PEQ}=\frac{{PEQ}_{i}\times IR\times EF\times ED}{AT}$$

The equivalent risk, determined using the RPF method (Risk_PEQ_), is calculated through Equation (6), where RfD_Ref_ represents the reference dose (RfD) for the reference chemical, PFOA.

(6) $${Risk}_{PEQ}=\frac{{ADD}_{PEQ}}{{RfD}_{Ref}}$$

Consequently, the aggregate equivalent risk for all PFASs, denoted as Total Risk_PEQ_, has been calculated using Equation (7), where Risk_PEQ-i_ signifies the equivalent risk for the i-th PFAS.

(7) $${Total\;Risk}_{PEQ}=\sum_{i=1}^{n}{Risk}_{PEQ-i}$$

#### 2.5.3. Comparison of Risks Assessed Under Different Approaches

The risks evaluated using the Hazard Index (HI) Approach and the Relative Potency Factor (RPF) Method were compared for both individual and total per- and polyfluoroalkyl substances (PFASs). In this context, the values of 0.1 and 1 are considered the acceptable thresholds for non-carcinogenic risk for individual and total PFASs, respectively [34,35].

### 2.6. Statistical and Uncertainty Analysis

Where applicable, the nonparametric Kruskal–Wallis H test and Mann–Whitney U test were conducted to compare different distributions. Moreover, Spearman’s correlation coefficient (ρ) was utilized for association analysis. All statistical analyses were performed using IBM^®^ SPSS^®^ Statistics software version 28 (IBM Company, Armonk, NY, USA). The significance level (α) was established at 0.05. To analyze the uncertainty of exposure and risk estimates, including ADD, HQ, and RPF-based PFOA-equivalent risks, 1,000,000 iterations of Monte Carlo simulations were conducted using Oracle Crystal Ball version 11.1.2.4 (Oracle Corporation, Austin, TX, USA). For this purpose, lognormal and triangular distributions were employed for the mass concentration of each PFAS (utilizing the mean and standard deviation of all samples) and the RPF for PFPeA, PFHpA, and PFDA (minimum = lower limit, as presented in Table 1; most probable = geometric mean of the lower and upper limits; maximum = upper limit, as presented in Table 1), respectively. For the water ingestion rate per unit body weight and water intake rate, the distributions were assumed based on the results of the KNHANES raw data.

## 3. Results

### 3.1. Temporal Changes in PFAS Concentrations

**Figure 2:**
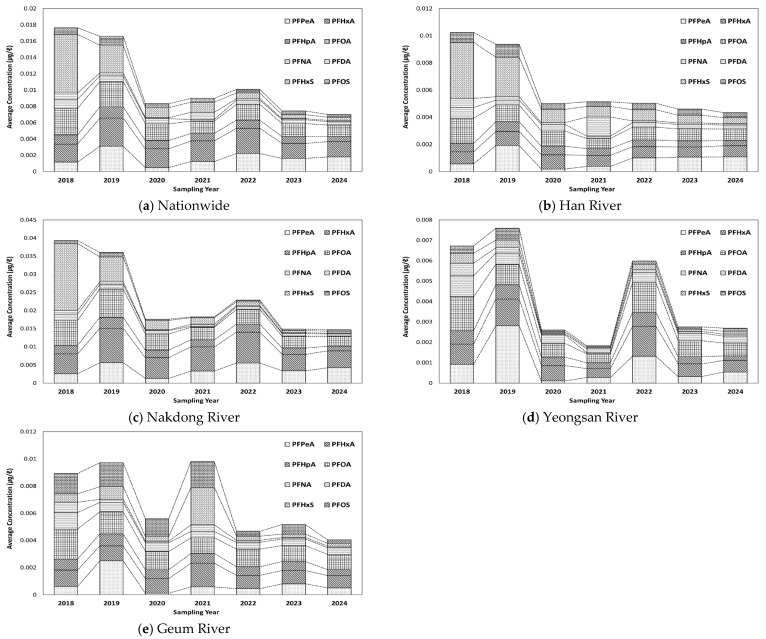
Temporal variations in PFAS concentrations in finished water on a nationwide scale and across river watersheds from 2018 to 2024.

A total of 1254 finished water samples were analyzed for the presence of eight targeted PFASs. All eight PFASs were detected in 288 samples, representing 23% of the total sample size. The detection frequencies of PFOA, PFHpA, PFHxA, PFNA, PFHxS, PFPeA, PFOS, and PFDA were 83%, 80%, 73%, 63%, 63%, 58%, 56%, and 52%, respectively. On the nationwide scale, the detection frequency did not vary significantly over the sampling years (Kruskal–Wallis H test, *p* = 0.423). Figure 2 illustrates the temporal changes in the average PFAS concentrations in the finished water samples from Korea.

Spearman’s correlation analysis indicates an overall decreasing linear trend in PFAS concentrations at the national level over the sampling years, with a similar trend observed across all river watersheds nationwide. Notably, significant reductions were observed in PFDA (average concentration: 0.0008 μg/L in 2018 to 0.0001 μg/L in 2024; 95th percentile: 0.0015 μg/L in 2018 to 0.0004 μg/L in 2024; ρ = −0.341 [95% CI: −0.391–−0.290]; *p* < 0.001), PFNA (average concentration: 0.0011 μg/L in 2018 to 0.0004 μg/L in 2024; 95th percentile: 0.0029 μg/L in 2018 to 0.0011 μg/L in 2024; ρ = −0.273 [95% CI: −0.325–−0.220]; *p* < 0.001), PFOA (average concentration: 0.0032 μg/L in 2018 to 0.0014 μg/L in 2024; 95th percentile: 0.0112 μg/L in 2018 to 0.0039 μg/L in 2024; ρ = −0.226 [95% CI: −0.279–−0.171]; *p* < 0.001), PFHxS (average concentration: 0.0072 μg/L in 2018 to 0.0004 μg/L in 2024; 95th percentile: 0.034 μg/L in 2018 to 0.001 μg/L in 2024; ρ = −0.177 [95% CI: −0.232–−0.121]; *p* < 0.001), and PFOS (average concentration: 0.0008 μg/L in 2018 to 0.0003 μg/L in 2024; 95th percentile: 0.002 μg/L in 2018 to 0.001 μg/L in 2024; ρ = −0.111 [95% CI: −0.167–−0.054]; *p* < 0.001).

The observed reduction in PFAS compounds, particularly PFOA, PFOS, and PFHxS, after implementing the monitoring standards in 2018 may indicate the efficacy of policies and interventions, such as regulatory measures or industrial modifications, which aimed to mitigate PFAS concentrations in potable water.

### 3.2. Reliability Evaluation of Hepatic RPFs

Table 3 summarizes the evaluation results on RPF reliability for each PFAS. The evaluation assessed data and study confidence to determine each PFAS compound’s overall reliability score. Data confidence, reflecting data quality and consistency, was rated high (7–10), medium (2–<7), and low (<2). Study confidence, evaluating methodologies and experimental robustness, was rated high (10–15), medium (5–<10), and low (<5). The overall reliability score combined data and study scores, classified as high (20–25), medium (10–<20), or low (<10). Overall reliability used absolute total-score thresholds; thus, combined medium sub-scores may result in low overall reliability if below 10.

It is significant to note that these overall thresholds were not derived by directly aggregating the sub-score categories; rather, they were based on absolute numerical values to reflect the asymmetric weightings inherent in the scoring scales (i.e., a maximum of 10-point vs. 15-point). This methodology was intentionally selected to maintain consistency and to avoid overestimating reliability in borderline cases. For example, even when both sub-scores fall within their respective “medium” categories, their combined score could still be below 10, indicating a conservative interpretation of overall reliability. The rationale for this threshold-based classification is elucidated in the Supplementary Materials (Tables S7–S14). Consequently, these categories denote the reliability of the RPFs for each PFAS, thereby facilitating the prioritization of compounds and justifying further investigation and confidence in their toxicity assessments.

Among the target PFASs evaluated, PFHxA had the highest reliability score, closely followed by PFHxS. These compounds demonstrated the most robust data and study confidence, reflecting a high level of consistency and reliability in available research.

For the PFAS for which both data and study confidence could be evaluated, the mean reliability score was 13.9, which falls within the medium category. This means that the RPF values for these PFASs were moderately reliable for this investigation. Although the mean score suggested a medium reliability level, it also highlighted the variability in the available data, indicating that some PFASs may require more extensive research or higher-quality data to improve their reliability scores.

### 3.3. Risk Analysis of PFASs in Finished Water Samples

**Figure 3:**
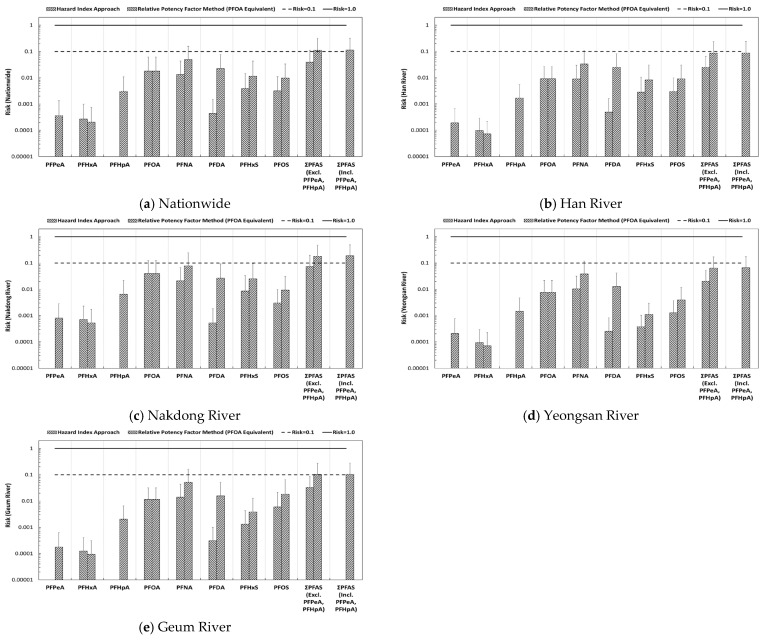
Results of hazard quotient for analyzed PFASs using individual toxicity and RPF method for finished water samples at the nationwide scale and drinking water treatment plants across river watersheds from 2018 to 2024 (error bars indicate the 95th percentile value).

Figure 3 illustrates risk assessment results for analyzed PFASs, employing individual toxicity and RPF methods, for finished water samples nationwide and at drinking water treatment plants in river watersheds from 2018 to 2024. Except for PFOA, the reference chemical, all PFASs exhibited statistically significant differences in risk values from the HI approach, based on individual toxicity, versus those derived from the RPF method, utilizing PFOA equivalents (Mann–Whitney U test, *p* < 0.001).

The total PFAS risk calculated via the RPF method (Total Risk_PEQ_) surpassed that determined using the HI approach. On the national scale, the disparity between Risk_PEQ_ and HQ ranged from −25% (for PFHxA) to approximately 5000% (for PFDA). This variability underscores the complexity of assessing the risk posed by PFAS mixtures in drinking water, as certain compounds demonstrate significantly greater toxic potential than others.

Regarding the distributions of risk calculated using both methods, the mean and 95th percentile values were below 1.0, indicating a low potential for non-carcinogenic health effects due to PFAS exposure in finished water. However, the 95th percentile value of Risk_PEQ_ for PFNA nationwide, as well as across all DWTPs in different watersheds, exceeded the acceptable threshold of 0.1. This finding suggests that vigilant monitoring of PFNA in finished waters throughout Korea is warranted.

## 4. Discussion

The results of this study can be compared with those of other nationwide studies. Research that has conducted surveys on the finished water quality directly from DWTPs is limited. However, various studies have measured tap water quality concerning PFASs.

Park et al. [22] investigated the presence of PFASs in tap water from eight major cities in South Korea. PFHxS, PFPeA, PFHxA, and PFOA were the most prevalent PFASs in water. Tap water derived from the Nakdong River, located in an industrial region, exhibited a higher proportion of PFHxS, which is consistent with the observations in the present study. These results were compared with the observations of the current study to provide an overview of the changes before and after the PFAS regulation implementation in Korea.

Teymoorian et al. [49] examined the presence of PFASs in tap water from various regions worldwide between 2021 and 2023. Seventy-six PFASs were screened in drinking water samples, primarily from Canada, USA, Europe, and Asia. The results revealed widespread PFAS contamination in tap water, with the most frequently detected compounds being perfluorobutane sulfonate (PFBS), PFOS, and perfluorobutanoic acid (PFBA). The results were used to compare the observations of the present study with measurements from Japan, the USA, Canada, and France.

As shown in Table 4, all target PFAS compounds measured from 2018 to 2024 in this study decreased compared to the 2017 survey. This reduction was notable for PFHxS (Park et al. [22]: 15.1 ng/L → this study: 2.34 ng/L, an 84% reduction). Despite differences in location, sampling methods, and survey durations, this study found significantly lower PFPeA concentrations in Korea (1.65 ng/L) than in Canada (10.7 ng/L) and France (12.5 ng/L) [49]. The decline in PFAS levels in Korea may result from stringent regulations. Korea established a water quality standard for PFOA, PFOS, and PFHxS in drinking water since 2018. To reduce PFASs in food, the government has increased regulations, banning PFASs in utensils, containers, and packaging from 2020 [50]. In the industrial sector, PFASs are regulated under Korea’s “POPs Control Act,” potentially reducing PFAS discharge into water bodies from industrial waste and wastewater [51]. The cross-country comparison in Table 4 was based on drinking- or tap-water PFAS concentration data harmonized with the exposure and risk-assessment assumptions used here. Accordingly, the included countries are representative of comparable datasets, not an exhaustive inventory of PFAS contamination. Data gaps for other countries should be interpreted cautiously, as they may reflect monitoring infrastructure, analytical capability, target PFAS coverage, sample matrices, reporting formats, and regulatory priorities rather than absence of contamination. Previous assessments indicate that PFAS contamination in drinking-water sources is widespread, while monitoring coverage remains uneven across regions [49,52]. A review of PFASs in potential drinking-water sources from 2014 to 2024 reported regional differences in contamination profiles and risk estimates, underscoring the need for harmonized monitoring in underrepresented regions [52]. Such efforts are essential for improving global comparability of PFAS exposure and mixture risk assessments.

The findings of this study should be interpreted in light of several limitations. The results were reported through a survey of finished water samples from DWTPs. However, the emergence of PFASs remains possible even after treatment because of the degradation of precursor compounds into PFASs [49,53]. Another significant consideration is that recent human epidemiology-based PFAS toxicity values may offer greater protection than earlier animal-based values and are highly pertinent for regulatory screening [23,24,25]. These values are derived from various critical effects, including immune response, developmental outcomes, serum lipids, cancer-related endpoints, and liver biomarkers. Their derivation and interpretation remain subjects of scientific debate, particularly concerning epidemiological uncertainty, biomarker-to-disease interpretation, dose–response assumptions, mixture additivity, and cross-agency differences in risk-assessment methodology [54]. Consequently, the present study retained a liver-focused framework to assess the applicability of hepatic RPFs and to facilitate an endpoint-consistent comparison with conventional hazard quotient calculations. Future research should investigate how alternative human-based toxicity values influence PFAS mixture risk estimates.

Another limitation of this study is the inclusion of only eight PFASs, which may lead to an underestimation of total PFAS exposure and cumulative risk, as additional PFAS and precursor compounds could be present in drinking water or other exposure media. Nonetheless, this restricted target list was a deliberate methodological choice, reflecting the study’s objective. The aim was not to conduct a comprehensive assessment of all PFAS exposure but to evaluate the applicability of a hepatic RPF-based mixture approach using PFASs for which nationwide monitoring data, hepatic RPFs, and compatible hepatic toxicity information were available. Including PFASs without endpoint-compatible RPFs or hepatic toxicity values would have broadened chemical coverage but compromised the internal consistency of the comparison between the RPF and HQ approaches. Future research should expand this framework as more PFAS-specific toxicity data, endpoint-specific RPFs, and monitoring data become available.

Despite these limitations, the results of the present study suggest that implementing monitoring policies for PFASs in drinking water since 2018 may have contributed to improved water quality in South Korea.

## 5. Conclusions

This study provides a long-term nationwide evaluation of PFASs in finished drinking water from major DWTPs across Korea. A key contribution is the use of an extensive monitoring dataset of 1254 finished water samples from 70 DWTPs collected over seven years, allowing an assessment of national temporal changes in PFAS concentrations. The reduction in several PFASs, notably PFOA, PFOS, and PFHxS, suggests that monitoring policies and management actions implemented since 2018 may have improved drinking-water quality. These findings highlight the need for ongoing nationwide monitoring to evaluate PFAS control measures and identify emerging or persistent compounds requiring further management.

Another major contribution is the application of an endpoint-specific hepatic mixture risk assessment framework using the RIVM relative potency factor (RPF) approach. By converting measured PFAS concentrations into PFOA-equivalent concentrations, the study shows how co-occurring PFASs in drinking water can be evaluated within a unified hepatic toxicity framework. Comparing the RPF-based and conventional hazard quotient-based approaches provides a basis for assessing whether single-compound evaluations may underestimate PFAS mixture risks. The higher cumulative risks estimated by the RPF method highlight the value of mixture-based assessment for drinking-water risk characterization.

This study also assesses the reliability of hepatic RPFs using a structured scoring system. This improves transparency in interpreting RPF-based risk estimates and helps identify PFASs needing additional toxicological data. Although the RIVM RPF method was suitable for this investigation, caution is needed for PFASs with lower confidence scores or RPFs derived by read-across. Further refinement of PFAS-specific toxicity data and endpoint-specific RPFs would strengthen future mixture risk assessments.

The findings should be viewed as an endpoint-specific evaluation of hepatic PFAS mixture assessment, not a comprehensive assessment of all PFAS-related health risks. Broader human-based guidance values may suit regulatory screening, but endpoint consistency remains crucial for evaluating and applying RPF-based approaches reliably.

## Figures and Tables

**Table 1:** The relative potency factor (RPF) for targeted PFASs.

No	Per- and Poly-Fluoroalkyl Substances (PFASs)	Chemical Abstracts Service Registry Number (CAS RN)	Relative Potency Factor (RPF) *	Remarks
1	Perfluoropentanoic acid (PFPeA)	2706-90-3	0.01–0.05	Read-across
2	Perfluorohexanoic acid (PFHxA)	307-24-4	0.01	Relative liver weight
3	Perfluoroheptanoic acid (PFHpA)	375-85-9	0.01–1	Read-across
4	Perfluorooctanoic acid (PFOA)	335-67-1	1.00	Relative liver weight
5	Perfluorononanoic acid (PFNA)	375-95-1	10	Relative liver weight
6	Perfluorodecanoic acid (PFDA)	335-76-2	4–10	Read-across
7	Perfluorohexanesulfonic acid (PFHxS)	355-46-4	0.6	Relative liver weight
8	Perfluorooctanesulfonic acid (PFOS)	1763-23-1	2	Relative liver weight

* The RPF values for each PFASs are those proposed by Bil et al. [27].

**Table 2:** The reference dose (RfD) for targeted PFASs regarding hepatic toxicity.

PFAS	Reference Dose (RfD) (μg/kg/day)	Reference for RfD	Remarks
PFPeA	N/A *	-	-
PFHxA	0.15	[38]	Hepatic system toxicity
PFHpA	N/A *	-	-
PFOA	0.002	[39]	Increase in relative liver weight
PFNA	0.00074	[40]	Increase in absolute and relative liver weight
PFDA	0.015	[41]	Increase in liver weight
PFHxS	0.00973	[42]	Increase in hepatic focal necrosis
PFOS	0.00307	[43]	Histological changes in the liver

* N/A: No reliable RfD for hepatic toxicity was available.

**Table 3:** Results of RPF Reliability Evaluation.

PFAS	RPF	Reference for Toxicity Data	Data Confidence		Study Confidence		Overall Reliability	
			Score	Evaluation	Score	Evaluation	Score	Evaluation
PFPeA	0.01–0.05	-	2.5	Medium	-	-	2.5	Low
PFHxA	0.01	[44]	2.5	Medium	14.1	High	16.6	Medium
PFHpA	0.01–1	-	1	Low	-	-	1	Low
PFOA	1.00	[45]	2.5	Medium	12.1	High	14.6	Medium
PFNA	10	[46]	2.5	Medium	9.9	Medium	12.4	Medium
PFDA	4–10	-	2.5	Medium	-	-	2.5	Low
PFHxS	0.6	[47]	2.5	Medium	14	High	16.5	Medium
PFOS	2	[48]	2.5	Medium	6.9	Medium	9.4	Low

**Table 4:** PFAS concentration and risk assessment in various nationwide studies.

PFAS	Country	Year	Samples	Analyzing Method	Average Concentration (ng/L)	Exposure (μg/kg/day) *		Risk *	
						Average Daily Dose (ADD)	PFOA Equivalent (ADD_PEQ_)	Hazard Quotient (HQ)	PFOA Equivalent (Risk_PEQ_)
PFPeA	Rep. of Korea	2018–2024 ^(1)^	Finished water	UPLC-MS/MS	1.63	2.65 × 10^−5^	5.92 × 10^−7^	-	2.96 × 10^−4^
		2017 ^(2)^	Tap water	HPLC-ESI/MS/MS	5.51	8.95 × 10^−5^	2.00 × 10^−6^		1.00 × 10^−3^
	Japan	2021–2022 ^(3)^		UHPLC/HRMS	4.03	6.55 × 10^−5^	1.46 × 10^−6^		7.32 × 10^−4^
	USA	2022–2023 ^(3)^			1.19	1.93 × 10^−5^	4.32 × 10^−7^		2.16 × 10^−4^
	Canada	2021–2023 ^(3)^			10.7	1.74 × 10^−4^	3.89 × 10^−6^		1.94 × 10^−3^
	France	2022–2023 ^(3)^			12.5	2.03 × 10^−4^	4.54 × 10^−6^		2.27 × 10^−3^
PFHxA	Rep. of Korea	2018–2024 ^(1)^	Finished water	UPLC-MS/MS	2.51	4.08 × 10^−5^	4.08 × 10^−7^	2.72 × 10^−4^	2.04 × 10^−4^
		2017 ^(2)^	Tap water	HPLC-ESI/MS/MS	5.52	8.97 × 10^−5^	8.97 × 10^−7^	5.98 × 10^−4^	4.48 × 10^−4^
	Japan	2021–2022 ^(3)^		UHPLC/HRMS	3.53	5.74 × 10^−5^	5.74 × 10^−7^	3.82 × 10^−4^	2.87 × 10^−4^
	USA	2022–2023 ^(3)^			1.23	2.00 × 10^−5^	2.00 × 10^−7^	1.33 × 10^−4^	9.99 × 10^−5^
	Canada	2021–2023 ^(3)^			6.80	1.10 × 10^−4^	1.10 × 10^−6^	7.37 × 10^−4^	5.52 × 10^−4^
	France	2022–2023 ^(3)^			17.1	2.78 × 10^−4^	2.78 × 10^−6^	1.85 × 10^−3^	1.39 × 10^−3^
PFHpA	Rep. of Korea	2018–2024 ^(1)^	Finished water	UPLC-MS/MS	1.01	1.64 × 10^−5^	1.64 × 10^−6^	-	8.21 × 10^−4^
		2017 ^(2)^	Tap water	HPLC-ESI/MS/MS	2.72	4.42 × 10^−5^	4.42 × 10^−6^		2.21 × 10^−3^
	Japan	2021–2022 ^(3)^		UHPLC/HRMS	1.59	2.58 × 10^−5^	2.58 × 10^−6^		1.29 × 10^−3^
	USA	2022–2023 ^(3)^			1.03	1.67 × 10^−5^	1.67 × 10^−6^		8.37 × 10^−4^
	Canada	2021–2023 ^(3)^			1.69	2.75 × 10^−5^	2.75 × 10^−6^		1.37 × 10^−3^
	France	2022–2023 ^(3)^			5.18	8.42 × 10^−5^	8.42 × 10^−6^		4.21 × 10^−3^
PFOA	Rep. of Korea	2018–2024 ^(1)^	Finished water	UPLC-MS/MS	2.20	3.57 × 10^−5^	3.57 × 10^−5^	1.79 × 10^−2^	1.79 × 10^−2^
		2017 ^(2)^	Tap water	HPLC-ESI/MS/MS	5.83	9.47 × 10^−5^	9.47 × 10^−5^	4.74 × 10^−2^	4.74 × 10^−2^
	Japan	2021–2022 ^(3)^		UHPLC/HRMS	2.77	4.50 × 10^−5^	4.50 × 10^−5^	2.25 × 10^−2^	2.25 × 10^−2^
	USA	2022–2023 ^(3)^			1.61	2.62 × 10^−5^	2.62 × 10^−5^	1.31 × 10^−2^	1.31 × 10^−2^
	Canada	2021–2023 ^(3)^			4.12	6.69 × 10^−5^	6.69 × 10^−5^	3.35 × 10^−2^	3.35 × 10^−2^
	France	2022–2023 ^(3)^			5.41	8.79 × 10^−5^	8.79 × 10^−5^	4.40 × 10^−2^	4.40 × 10^−2^
PFNA	Rep. of Korea	2018–2024 ^(1)^	Finished water	UPLC-MS/MS	0.60	9.75 × 10^−6^	9.75 × 10^−5^	1.32 × 10^−2^	4.87 × 10^−2^
		2017 ^(2)^	Tap water	HPLC-ESI/MS/MS	0.87	1.41 × 10^−5^	1.41 × 10^−4^	1.91 × 10^−2^	7.07 × 10^−2^
	Japan	2021–2022 ^(3)^		UHPLC/HRMS	1.13	1.84 × 10^−5^	1.84 × 10^−4^	2.48 × 10^−2^	9.18 × 10^−2^
	USA	2022–2023 ^(3)^			0.34	5.52 × 10^−6^	5.52 × 10^−5^	7.47 × 10^−3^	2.76 × 10^−2^
	Canada	2021–2023 ^(3)^			0.31	5.04 × 10^−6^	5.04 × 10^−5^	6.81 × 10^−3^	2.52 × 10^−2^
	France	2022–2023 ^(3)^			0.57	9.26 × 10^−6^	9.26 × 10^−5^	1.25 × 10^−2^	4.63 × 10^−2^
PFDA	Rep. of Korea	2018–2024 ^(1)^	Finished water	UPLC-MS/MS	0.41	6.66 × 10^−6^	4.21 × 10^−5^	4.44 × 10^−4^	2.11 × 10^−2^
		2017 ^(2)^	Tap water	HPLC-ESI/MS/MS	0.44	7.15 × 10^−6^	4.52 × 10^−5^	4.77 × 10^−4^	2.26 × 10^−2^
	Japan	2021–2022 ^(3)^		UHPLC/HRMS	0.08	1.30 × 10^−6^	8.22 × 10^−6^	8.67 × 10^−5^	4.11 × 10^−3^
	USA	2022–2023 ^(3)^			0.07	1.14 × 10^−6^	7.19 × 10^−6^	7.58 × 10^−5^	3.60 × 10^−3^
	Canada	2021–2023 ^(3)^			0.06	9.75 × 10^−7^	6.17 × 10^−6^	6.50 × 10^−5^	3.08 × 10^−3^
	France	2022–2023 ^(3)^			0.08	1.30 × 10^−6^	8.22 × 10^−6^	8.67 × 10^−5^	4.11 × 10^−3^
PFHxS	Rep. of Korea	2018–2024 ^(1)^	Finished water	UPLC-MS/MS	2.34	3.80 × 10^−5^	2.28 × 10^−5^	3.91 × 10^−3^	1.14 × 10^−2^
		2017 ^(2)^	Tap water	HPLC-ESI/MS/MS	15.1	2.45 × 10^−4^	1.47 × 10^−4^	2.52 × 10^−2^	7.36 × 10^−2^
	Japan	2021–2022 ^(3)^		UHPLC/HRMS	0.46	7.47 × 10^−6^	4.48 × 10^−6^	7.68 × 10^−4^	2.24 × 10^−3^
	USA	2022–2023 ^(3)^			0.29	4.71 × 10^−6^	2.83 × 10^−6^	4.84 × 10^−4^	1.41 × 10^−3^
	Canada	2021–2023 ^(3)^			1.56	2.53 × 10^−5^	1.52 × 10^−5^	2.61 × 10^−3^	7.60 × 10^−3^
	France	2022–2023 ^(3)^			3.24	5.26 × 10^−5^	3.16 × 10^−5^	5.41 × 10^−3^	1.58 × 10^−2^
PFOS	Rep. of Korea	2018–2024 ^(1)^	Finished water	UPLC-MS/MS	0.60	9.77 × 10^−6^	1.96 × 10^−5^	3.19 × 10^−3^	9.77 × 10^−3^
		2017 ^(2)^	Tap water	HPLC-ESI/MS/MS	0.71	1.15 × 10^−5^	2.31 × 10^−5^	3.76 × 10^−3^	1.15 × 10^−2^
	Japan	2021–2022 ^(3)^		UHPLC/HRMS	0.56	9.10 × 10^−6^	1.82 × 10^−5^	2.96 × 10^−3^	9.10 × 10^−3^
	USA	2022–2023 ^(3)^			0.91	1.48 × 10^−5^	2.96 × 10^−5^	4.82 × 10^−3^	1.48 × 10^−2^
	Canada	2021–2023 ^(3)^			1.63	2.65 × 10^−5^	5.30 × 10^−5^	8.63 × 10^−3^	2.65 × 10^−2^
	France	2022–2023 ^(3)^			3.07	4.99 × 10^−5^	9.98 × 10^−5^	1.62 × 10^−2^	4.99 × 10^−2^

* Calculated for average concentrations based on the assumptions of this study. ^(1)^ This study; ^(2)^ [22]; ^(3)^ [49]. PFPeA: Perfluoropentanoic acid; PFHxA: Perfluorohexanoic acid; PFHpA: Perfluoroheptanoic acid; PFOA: Perfluorooctanoic acid; PFNA: Perfluorononanoic acid; PFDA: Perfluorodecanoic acid; PFHxS: Perfluorohexanesulfonic acid; PFOS: Perfluorooctanesulfonic acid.

## Data Availability

The data supporting the findings of this study are provided in the article and Supplementary Materials.

## Supplementary Materials

The following supporting information can be downloaded at: https://www.mdpi.com/article/10.3390/toxics14070577/s1, Figure S1: Sample Preparation Procedure of PFAS Analysis; Figure S2: UPLC-MS/MS Extract Ion Chromatograms of (a) Blank and (b) Spiked PFAS (50 ng/L); Table S1: UPLC Operating Conditions for PFAS; Table S2: MS/MS Operating Conditions for PFAS; Table S3: Typical Standard Calibration Data and Detection Limits of PFAS; Table S4: Results of Accuracy and Precision of PFAS (n = 5); Table S5: Annual Sample Size in Nationwide and River Watersheds; Table S6: The Reliability Scoring System for Toxicity Data; Table S7: The RPF Reliability Evaluation for PFPeA; Table S8: The RPF Reliability Evaluation for PFHxA; Table S9: The RPF Reliability Evaluation for PFHpA; Table S10: The RPF Reliability Evaluation for PFOA; Table S11: The RPF Reliability Evaluation for PFNA; Table S12: The RPF Reliability Evaluation for PFDA; Table S13: The RPF Reliability Evaluation for PFHxS; Table S14: The RPF Reliability Evaluation for PFOS.

## Abbreviations

The following abbreviations are used in this manuscript:

ADD	Average daily dose
ADD_PEQ_	PFOA-equivalent average daily dose
APFO	Ammonium perfluorooctanoate
AT	Average time of exposure
BMD	Benchmark dose
BMDL	Benchmark dose lower confidence limit
BW	Body weight
CAS RN	Chemical Abstracts Service Registry Number
CI	Confidence interval
DWTP	Drinking water treatment plant
ED	Exposure duration
EF	Exposure frequency
ECHA	European Chemicals Agency
EPA	Environmental Protection Agency
GLP	Good laboratory practice
HFPO-DA	Hexafluoropropylene oxide dimer acid
HI	Hazard index
HLB	Hydrophilic–lipophilic balance
HPLC-ESI/MS/MS	High-performance liquid chromatography–electrospray ionization tandem mass spectrometry
HQ	Hazard quotient
IR	Ingestion rate
IRIS	Integrated Risk Information System
K-REACH	Act on the Registration and Evaluation of Chemicals
KNHANES	Korea National Health and Nutrition Examination Survey
LC	Liquid chromatography
MCL	Maximum contaminant level
MDL	Method detection limit
MeOH	Methanol
MOE	Ministry of Environment
MS/MS	Tandem mass spectrometry
N/A	Not applicable/not available
NIER	National Institute of Environmental Research
OPFRs	Organophosphate flame retardants
PEQ	PFOA-equivalent concentration
PFAC-MXC	PFAS mixed calibration solution
PFAS	Per- and polyfluoroalkyl substances
PFBA	Perfluorobutanoic acid
PFBS	Perfluorobutane sulfonate
PFDA	Perfluorodecanoic acid
PFHpA	Perfluoroheptanoic acid
PFHxA	Perfluorohexanoic acid
PFHxS	Perfluorohexanesulfonic acid
PFNA	Perfluorononanoic acid
PFOA	Perfluorooctanoic acid
PFOS	Perfluorooctanesulfonic acid
PFPeA	Perfluoropentanoic acid
POD	Point of departure
POPs	Persistent organic pollutants
PP	Polypropylene
RfD	Reference dose
RfD_Ref_	Reference dose for the reference chemical
RIVM	National Institute for Public Health and the Environment
RPF	Relative potency factor
SD	Standard deviation
SDGs	Sustainable Development Goals
SPE	Solid-phase extraction
TCEQ	Texas Commission on Environmental Quality
TDI	Tolerable daily intake
UHPLC/HRMS	Ultra-high-performance liquid chromatography/high-resolution mass spectrometry
UPLC	Ultra-performance liquid chromatography
UPLC-MS/MS	Ultra-performance liquid chromatography coupled with tandem mass spectrometry
US-EPA	United States Environmental Protection Agency
