# Supplementary material for: Nationwide Monitoring and Hepatic Mixture Risk Assessment of PFASs in Korean Drinking Water Using Relative Potency Factors
Source: Toxics. 2026 Jun 30;14(7):577. doi: 10.3390/toxics14070577 (PMC13417505; doi:10.3390/toxics14070577)
Supplement: Supplementary file 1 [file toxics-14-00577-s001.zip › toxics-4372187-supplementary.pdf]

toxics

MDPI

Supplementary Materials

Nationwide Monitoring and Hepatic Mixture Risk Assessment of PFASs in Korean Drinking Water Using Relative Potency Factors

Yubeen Kim [1,2,3], Shervin Hashemi [4], Heesoo Pyo [5], Youngwook Lim [4,6], Changsoo Kim [4,6], Incheol Choi [7] and Jiyeon Yang [1,4,*]

1. Graduate School of Public Health, Yonsei University, 50-1 Yonsei-ro, Seodaemun-gu, Seoul 03722, Republic of Korea; kyb8542@kosha.or.kr
2. Inhalation Toxicity Research Center, Occupational Safety and Health Research Institute, Korea Occupational Safety and Health Agency , Daejeon 34122 , Republic of Korea
3. College of Pharmacy, Dankook University, Cheonan-si 31116 , Republic of Korea
4. Institute for Environmental Research, Yonsei University College of Medicine, 50-1 Yonsei-ro, Seodaemun-gu, Seoul 03722, Republic of Korea; shervin@yuhs.ac (S.H.); envlim@yuhs.ac (Y.L.); preman@yuhs.ac (C.K.)
5. International Advanced Analysis Institute, B-339, 140, Tongil-ro, Deogyang-gu, Goyang-si, Gyeonggi-do 10594, Republic of Korea; phs3692@kist.re.kr
6. Department of Preventive Medicine and Public Health, Yonsei University College of Medicine, 50-1 Yonsei-ro, Seodaemun-gu, Seoul 03722, Republic of Korea
7. Water Use & Management Division, National Institute of Environmental Research, Hwangyong-ro 42, Seogu, Incheon 22689, Republic of Korea; cic00@korea.kr

* Correspondence: jyyang67@yuhs.ac; Tel.: +82-2-2228-1896

Contents

Figure S1. Sample Preparation Procedure of PFAS Analysis 1

Table S1. UPLC Operating Conditions for PFAS 2

Table S2. MS/MS Operating Conditions for PFAS 2

Figure S2. UPLC-MS/MS Extract Ion Chromatograms of (a) Blank and (b) Spiked PFAS (50 ng/L) 3

Table S3. Typical Standard Calibration Data and Detection Limits of PFAS 4

Table S4. Results of Accuracy and Precision of PFAS (n = 5) 4

Table S5. Annual Sample Size in Nationwide and River Watersheds 5

Table S6. The Reliability Scoring System for Toxicity Data 6

Table S7. The RPF Reliability Evaluation for PFPeA 7

Table S8. The RPF Reliability Evaluation for PFHxA 8

Table S9. The RPF Reliability Evaluation for PFHpA 9

Table S10. The RPF Reliability Evaluation for PFOA 10

Table S11. The RPF Reliability Evaluation for PFNA 11

Table S12. The RPF Reliability Evaluation for PFDA 12

Table S13. The RPF Reliability Evaluation for PFHxS 13

Table S14. The RPF Reliability Evaluation for PFOS 14

References 15

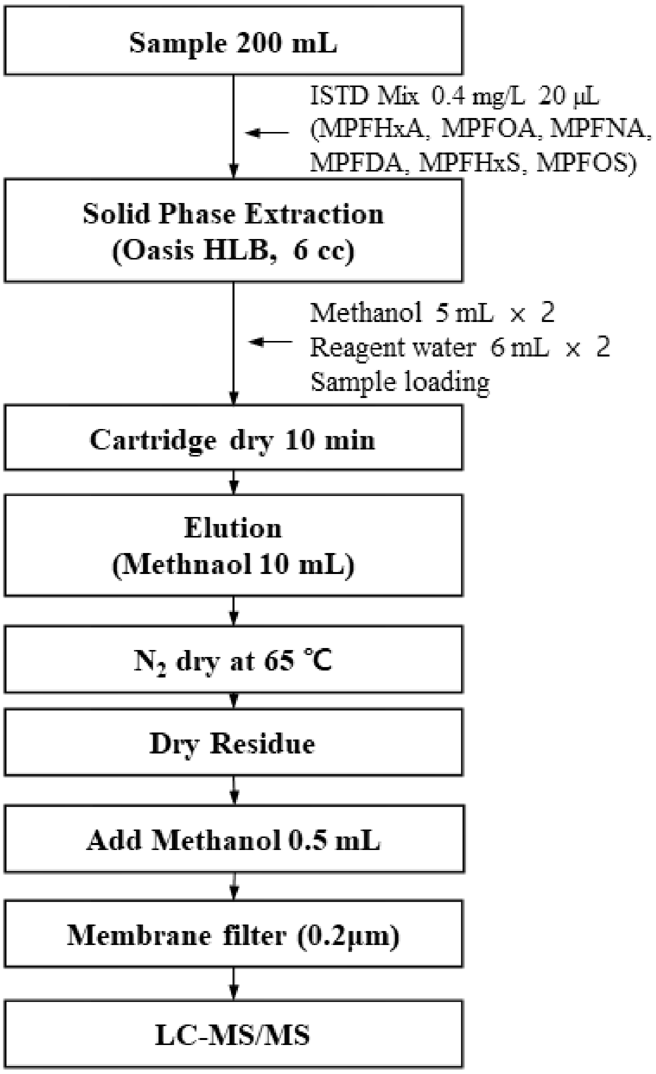

Figure S1. Sample Preparation Procedure of PFAS Analysis

Table S1. UPLC Operating Conditions for PFAS

· **Column :** Hypersil GOLD™ C18 Selectivity LC Columns (2.1 mm, 100 mm length, 1.9 μm particle size)
· **Column Oven Temp. :** 40 ℃
· **Buffer A :** 5 mM ammonium acetate in water
· **Buffer B :** MeOH
· **Flow rate :** 0.3 mL/min (constant flow)
· **Injection Volume :** 5 μL
· **Run Time :** 20 min
· **UPLC Gradient Condition :**

Time (min)	Buffer A (%)	Buffer B (%)
0	90	10
0.50	90	10
1.00	40	60
10.0	5	95
14.0	5	95
15.5	90	10
23.0	90	10

Table S2. MS/MS Operating Conditions for PFAS

· **Ionization Mode :** Negative ion electrospray
· **Capillary Voltage :** 3.50 kV
· **Vaporizer Temperature :** 325℃
· **Sheath Gas :** 40 Arb
· **Aux Gas :** 10 Arb
· **Sweep Gas :** 1 Arb
· **Acquisition mode :** MRM (Multiple Reaction Monitoring) Mode
· **MRM Conditions :**

Compound	Precursor Ion (m/z)	Product ion 1 (m/z) (CE*)	Product ion 2 (m/z) (CE)	RF Lens (volt)
PFPeA	262.912	219 (8.16)	262.912 (1)	75
PFHxA	312.962	269.071 (8.41)	119 (19.79)	83
PFHpA	362.962	319.071 (9.04)	169 (16.63)	92
PFOA	412.962	369.071 (9.34)	169 (17.43)	101
PFNA	462.962	419 (9.47)	169 (18.61)	112
PFDA	512.962	469 (10.27)	269 (16.79)	121
PFHxS	398.962	80 (38.15)	98.929 (35.2)	223
PFOS	498.962	80 (41.9)	98.929 (41.69)	200
MPFHxA	315	270 (9)	315 (1)	82
MPFHxS	403	84 (38)	103 (35)	224
MPFOA	417	372 (7)	169 (17)	109
MPFNA	468	423 (9)	219 (15)	113
MPFOS	502.95	80.018 (41.86)	99.018 (41.65)	200
MPFDA	515	470 (12)	515 (1)	118

* CE : Collision Energy

(a) Blank

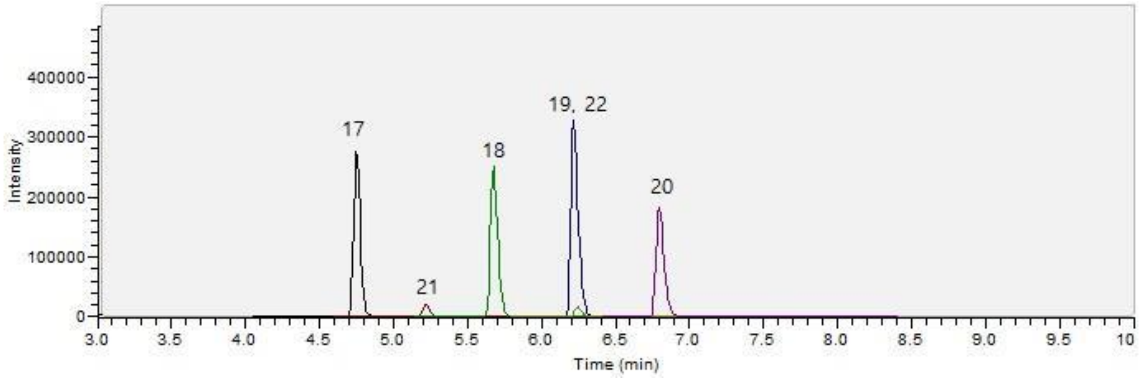

(b) Spiked

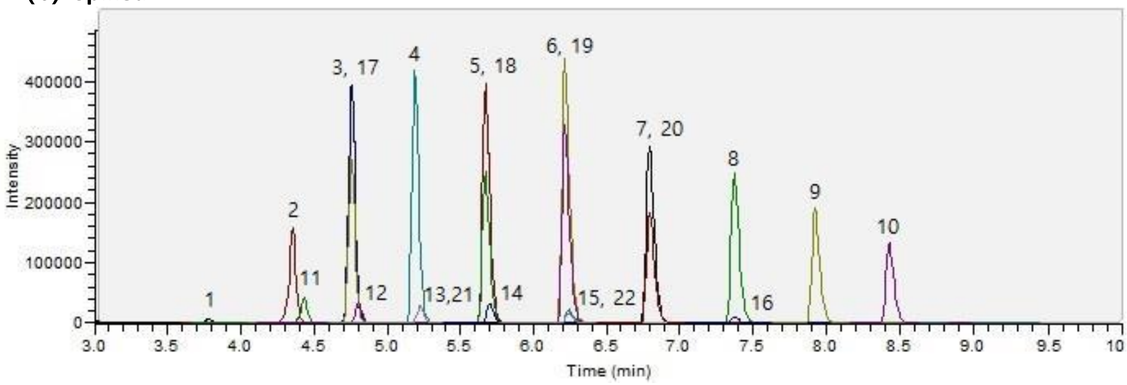

Peak Identity

Peak No.	Compounds	Retention Time (min)	Precursor Ion (m/z)	Product ion 1 (m/z) (CE**)	Product ion 2 (m/z) (CE)	RF Lens (volt)
**ISTD* (17)**	MPFHxA	5.16	315	270 (9)	315 (1)	82
**ISTD (21)**	MPFHxS	5.73	403	84 (38)	103 (35)	224
**ISTD (18)**	MPFOA	6.31	417	372 (7)	169 (17)	109
**ISTD (19)**	MPFNA	6.97	468	423 (9)	219 (15)	113
**ISTD (22)**	MPFOS	6.99	502.95	80.018 (41.86)	99.018 (41.65)	200
**ISTD (20)**	MPFDA	7.63	515	470 (12)	515 (1)	118
**2**	PFPeA	4.66	262.912	219 (8.16)	262.912 (1)	75
**3**	PFHxA	5.16	312.962	269.071 (8.41)	119 (19.79)	83
**4**	PFHpA	5.69	362.962	319.071 (9.04)	169 (16.63)	92
**5**	PFOA	6.31	412.962	369.071 (9.34)	169 (17.43)	101
**6**	PFNA	6.97	462.962	419 (9.47)	169 (18.61)	112
**7**	PFDA	7.63	512.962	469 (10.27)	269 (16.79)	121
**13**	PFHxS	5.73	398.962	80 (38.15)	98.929 (35.2)	223
**15**	PFOS	6.99	498.962	80 (41.9)	98.929 (41.69)	200

* ISTD : Internal Standard
** CE : Collision Energy

Figure S2. UPLC-MS/MS Extract Ion Chromatograms of (a) Blank and (b) Spiked PFAS (50 ng/L)

Table S3. Typical Standard Calibration Data and Detection Limits of PFAS

Compound	Syringe ISTD*	ISTD		Selected Ion (m/z)	Concentration Range (ng/L)			r^2	2018-2024 MDL** Range (ng/L)	2018-2024 PQL** Range (ng/L)
		2018	2019-2024		2018	2019-2021	2022-2024			
PFPeA	$^{13}C_8$-PFOA	$^{13}C_4$-PFOA	$^{13}C_2$-PFHxA	262.7	0.1 – 200	0.1 – 100	0.5 – 100	> 0.999	0.2 – 0.4	0.6 − 1.3
PFHxA				312.9	0.1 – 200	0.1 – 100	0.5 – 100	> 0.999	0.2 – 0.5	0.5 − 1.7
PFHpA			$^{13}C_4$-PFOA	362.8	0.1 – 200	0.1 – 100	0.5 – 100	> 0.999	0.2 – 0.3	0.5 − 1.1
PFOA				412.9	0.1 – 200	0.1 – 100	0.5 – 100	> 0.999	0.1 – 0.4	0.5 − 1.3
PFNA			$^{13}C_5$-PFNA	463	0.1 – 200	0.1 – 100	0.5 – 100	> 0.999	0.1 – 0.4	0.3 − 1.3
PFDA			$^{13}C_2$-PFDA	513	0.1 – 200	0.1 – 100	0.5 – 100	> 0.999	0.1 – 0.2	0.4 − 0.8
PFHxS	$^{13}C_8$-PFOS	$^{13}C_4$-PFOS	$^{18}O_2$-PFHxS	398.8	0.1 – 190	0.1 – 95	0.5 – 95	> 0.999	0.1 – 0.3	0.3 − 0.9
PFOS			$^{13}C_4$-PFOS	498.9	0.1 – 192	0.1 – 96	0.5 – 96	> 0.999	0.1 – 0.2	0.5 − 1.4

MDL: Method Detection Limit; Practical Quantitation Limit

* Syringe ISTD was not applied in 2018.

** MDL: Standard Deviation × 3.14; PQL: Standard Deviation × 10.0

Table S4. Results of Accuracy and Precision of PFAS (n = 5)

Compound	Low-High Spiked Concentration (ng/L)			2018-2024 Accuracy Range (%)		2018-2024 Precision Range (% CV)	
	2018-2019	2020-2021	2022-2024	Low Spiked Conc.	High Spiked Conc.	Low Spiked Conc.	High Spiked Conc.
PFPeA	2 – 10	2 – 20	10 – 50	85.1 − 99	82.4 − 98.3	3 − 9.6	1.1 − 7.1
PFHxA	2 – 10	2 – 20	10 – 50	92.2 − 103	87 − 99.2	1.2 − 5	0.7 − 5.1
PFHpA	2 – 10	2 – 20	10 – 50	89.4 − 104	86.9 − 103	2.1 − 6.6	1.2 − 5.5
PFOA	2 – 10	2 – 20	10 – 50	93.6 − 104	85.7 − 98	1.1 − 6.9	1.4 − 10.8
PFNA	2 – 10	2 – 20	10 – 50	93.7 − 102	87.4 − 102	1.6 − 8.7	1.1 − 6.6
PFDA	2 – 10	2 – 20	10 – 50	94.4 − 99.4	79.6 − 99.8	1.5 − 6.7	1.4 − 8.7
PFHxS	2 – 9	2 – 19	9 – 47	83.9 − 94.6	88.7 − 96.7	2.5 − 6.4	1.2 − 4.7
PFOS	2 – 10	2 – 19	10 – 48	87.5 − 101	89.9 − 101	2.1 − 6.2	0.5 − 9.5

Table S5. Annual Sample Size in Nationwide and River Watersheds

Scale / River Watershed	Sample Size							
	**Total**	**2018**	**2019**	**2020**	**2021**	**2022**	**2023**	**2024**
Nationwide*	1254	206	210	209	210	140	140	139
Han River	552	93	93	93	93	60	60	60
Nakdong River	357	58	60	60	60	40	40	39
Yeongsan River	129	22	24	23	24	12	12	12
Geum River	186	27	27	27	27	26	26	26

* Including all watersheds and samples from Jeju Special Self-Governing Province

Table S6. The Reliability Scoring System for Toxicity Data

Classification 1	Classification 2	Classification 3	Content	Score
Data Confidence (DC)	(DC-Criterion 1) Confidence in the determination of toxicity values		The determination of toxicity values was peer-reviewed.	5
			Else	0
	(DC-Criterion 2) Performing benchmark dose (BMD) modeling		The difference between BMD and BMDL is less than two-fold.	2.5
			Else	1
	(DC-Criterion 3) Evaluating substances with read-across applied		The equivalent PFOA concentration range using the RPF limits falls within the range determined by the geometric mean ±1SD of the PFOA equivalent distribution.	2.5
			The equivalent PFOA concentration range using the RPF limits falls within the range determined by the geometric mean ±2SD of the PFOA equivalent distribution.	1.5
			The equivalent PFOA concentration range using the RPF limits falls within or over the range determined by the geometric mean ±3SD of the PFOA equivalent distribution.	1
Study Confidence (SC)	(SC-Criterion 1) Good laboratory practice (GLP) compliance status		It complies with the Organization for Economic Co-operation and Development (OECD) or specific country standard testing guidelines.	4
			Else	0
	(SC-Criterion 2) Adequacy of toxicity testing	Test species	Human epidemiological study	1
			Primate	0.8
			Animal (mouse, rat, etc.)	0.5
		Exposure duration	Chronic (more than 1.5 years)	1
			Subchronic (90 days to more than 1.5 years)	0.8
			Subacute (28 to 89 days)	0.5
			Acute (less than 28 days)	0.3
		Administration method - Oral administration	Gavage	1
			Dietary: drinking water	0.5
			Dietary: capsule or fed	0.2
		Number of animals and sex distribution	Over 10 per group per male and female	1
			Over 10 per group per male or female	0.8
			3 – 9 per group per male and female	0.6
			3 – 9 per group per male or female	0.4
		Test type	Repeated dose toxicity study with a 2-generation reproductive and developmental toxicity study	1
			Repeated dose toxicity study	0.8
			Single-dose toxicity study	0
		Preliminary dose setting study	Performed	1
			Not performed	0
	(SC-Criterion 3) Substance purity		Over 95% purity	5
			85–95 purity	3
			Mixed, commercial score, purity not specified	1

Table S7. The RPF Reliability Evaluation for PFPeA

Classification 1	Classification 2	Classification 3	Content	Score	Remarks	Reference
Data Confidence	Confidence in the determination of toxicity values		Else	0	Read-across	
	Performing benchmark dose (BMD) modeling		-	0		
	Evaluating substances with read-across applied		The equivalent PFOA concentration range using the RPF limits falls within the range determined by the geometric mean ±1SD of the PFOA equivalent distribution	2.5	Equivalent PFOA concentration range using the RPF limits: 1.63×10^{-5} – 8.13×10^{-5} Range determined by the geometric mean ±1SD: -3.47×10^{-5} – 1.07×10^{-4}	This Study
Study Confidence	Good laboratory practice (GLP) compliance status		Else	0	Read-across	
	Adequacy of toxicity testing	Test species	-	0		
		Exposure duration	-	0		
		Administration method - Oral administration	-	0		
		Number of animals and sex distribution	-	0		
		Test type	-	0		
		Preliminary dose setting study	Not performed	0		
	Substance purity		-	0		

Table S8. The RPF Reliability Evaluation for PFHxA

Classification 1	Classification 2	Classification 3	Content	Score	Remarks	Reference
Data Confidence	Confidence in the determination of toxicity values		Else	0	There was no mention of it.	Bil et al. [27]
	Performing benchmark dose (BMD) modeling		The difference between BMD and BMDL is less than 2-fold	2.5	BMD(5) = 27 mg/kg/day; BMDL(5) = 19 mg/kg/day	
	Evaluating substances with read-across applied		-	0	Not applicable	
Study Confidence	Good laboratory practice (GLP) compliance status		It complies with the Organization for Economic Co-operation and Development (OECD) or specific country standard testing guidelines	4	OECD Guideline 408 (Repeated Dose 90-Day Oral Toxicity Study in Rodents)	Loveless et al. [44]
	Adequacy of toxicity testing	Test species	Animal (mouse, rat, etc.)	0.5	Sprague Dawley rats	
		Exposure duration	Subchronic (90 days to more than 1.5 years)	0.8	90 days	
		Administration method - Oral administration	Gavage	1		
		Number of animals and sex distribution	Over 10 per group per male and female	1	Male and Female, 10 per dose group	
		Test type	Repeated dose toxicity study	0.8		
		Preliminary dose setting study	Performed	1	Dosages were set at 20, 100, and 500 mg/kg/day, based on range finder studies.	
	Substance purity		Over 95% purity	5	White solid, 100% purity NaPFHx (CAS RN 2923-26-4) diluted in nano-pure water.	

Table S9. The RPF Reliability Evaluation for PFHpA

Classification 1	Classification 2	Classification 3	Content	Score	Remarks	Reference
Data Confidence	Confidence in the determination of toxicity values		Else	0	Read-across	
	Performing benchmark dose (BMD) modeling		-	0		
	Evaluating substances with read-across applied		The equivalent PFOA concentration range using the RPF limits falls within the range determined by the geometric mean ±3SD of the PFOA equivalent distribution	1	Equivalent PFOA concentration range using the RPF limits: 1.01×10^{-5} – 1.01×10^{-3} Range determined by the geometric mean ±3SD: -2.77×10^{-4} – 4.79×10^{-4}	This Study
Study Confidence	Good laboratory practice (GLP) compliance status		Else	0	Read-across	
	Adequacy of toxicity testing	Test species	-	0		
		Exposure duration	-	0		
		Administration method - Oral administration	-	0		
		Number of animals and sex distribution	-	0		
		Test type	-	0		
		Preliminary dose setting study	Not performed	0		
	Substance purity		-	0		

Table S10. The RPF Reliability Evaluation for PFOA

Classification 1	Classification 2	Classification 3	Content	Score	Remarks	Reference
Data Confidence	Confidence in the determination of toxicity values		Else	0	There was no mention of it.	Bil et al. [27]
	Performing benchmark dose (BMD) modeling		The difference between BMD and BMDL is less than 2-fold	2.5	BMD(5) = 0.3 mg/kg/day; BMDL(5) = 0.21 mg/kg/day	
	Evaluating substances with read-across applied		-	0	Not applicable	
Study Confidence	Good laboratory practice (GLP) compliance status		It complies with the Organization for Economic Co-operation and Development (OECD) or specific country standard testing guidelines	4	OECD reproductive/developmental toxicity	Perkins et al. [45]
	Adequacy of toxicity testing	Test species	Animal (mouse, rat, etc.)	0.5	Sprague Dawley rats	
		Exposure duration	Subchronic (90 days to more than 1.5 years)	0.8	91 days	
		Administration method - Oral administration	Dietary: capsule or fed	0.2	A well-mixed diet of 200g was prepared with the addition of APFO, and the homogeneity of APFO concentration in the diet was measured.	
		Number of animals and sex distribution	Over 10 per group per male or female	0.8	Male, 15 per dose group	
		Test type	Repeated dose toxicity study	0.8		
		Preliminary dose setting study	Not performed	0	There was no mention of it.	
	Substance purity		Over 95% purity	5	APFO 98.0% pure in fed	

Table S11. The RPF Reliability Evaluation for PFNA

Classification 1	Classification 2	Classification 3	Content	Score	Remarks	Reference
Data Confidence	Confidence in the determination of toxicity values		Else	0	There was no mention of it.	Bil et al. [27]
	Performing benchmark dose (BMD) modeling		The difference between BMD and BMDL is less than 2-fold	2.5	BMD(5) = 0.022 mg/kg/day; BMDL(5) = 0.016 mg/kg/day	
	Evaluating substances with read-across applied		-	0	Not applicable	
Study Confidence	Good laboratory practice (GLP) compliance status		It complies with the Organization for Economic Co-operation and Development (OECD) or specific country standard testing guidelines	4	The study was conducted under GLP practices, and no deviations that could impact the quality or interpretation of the results occurred.	Mertens et al. [46]
	Adequacy of toxicity testing	Test species	Animal (mouse, rat, etc.)	0.5	Sprague Dawley rats	
		Exposure duration	Subchronic (90 days to more than 1.5 years)	0.8	91 days	
		Administration method - Oral administration	Gavage	1		
		Number of animals and sex distribution	3 – 9 per group per male and female	0.6	Male and Female, 5 per dose group	
		Test type	Repeated dose toxicity study with a 2-generation reproductive and developmental toxicity study	1		
		Preliminary dose setting study	Performed	1	The selected dose levels, based on the results of the OECD 422 reproductive/developmental toxicity screening study, were 0.025, 0.125, and 0.6 mg/kg/day.	
	Substance purity		Mixed, commercial score, purity not specified	1		

Table S12. The RPF Reliability Evaluation for PFDA

Classification 1	Classification 2	Classification 3	Content	Score	Remarks	Reference
Data Confidence	Confidence in the determination of toxicity values		Else	0	Read-across	
	Performing benchmark dose (BMD) modeling		-	0		
	Evaluating substances with read-across applied		The equivalent PFOA concentration range using the RPF limits falls within the range determined by the geometric mean ±1SD of the PFOA equivalent distribution	2.5	Equivalent PFOA concentration range using the RPF limits: 1.63×10^{-3} – 4.06×10^{-3} Range determined by the geometric mean ±1SD: -9.69×10^{-3} – 1.48×10^{-2}	This Study
Study Confidence	Good laboratory practice (GLP) compliance status		Else	0	Read-across	
	Adequacy of toxicity testing	Test species	-	0		
		Exposure duration	-	0		
		Administration method - Oral administration	-	0		
		Number of animals and sex distribution	-	0		
		Test type	-	0		
		Preliminary dose setting study	Not performed	0		
	Substance purity		-	0		

Table S13. The RPF Reliability Evaluation for PFHxS

Classification 1	Classification 2	Classification 3	Content	Score	Remarks	Reference
Data Confidence	Confidence in the determination of toxicity values		Else	0	There was no mention of it.	Bil et al. [27]
	Performing benchmark dose (BMD) modeling		The difference between BMD and BMDL is less than 2-fold	2.5	BMD(5) = 0.51 mg/kg/day; BMDL(5) = 0.37 mg/kg/day	
	Evaluating substances with read-across applied		-	0	Not applicable	
Study Confidence	Good laboratory practice (GLP) compliance status		It complies with the Organization for Economic Co-operation and Development (OECD) or specific country standard testing guidelines	4	OECD 422 guideline-based design	Butenhoff et al. [47]
	Adequacy of toxicity testing	Test species	Animal (mouse, rat, etc.)	0.5	Sprague Dawley rats	
		Exposure duration	Subacute (28 to 89 days)	0.5	42 days	
		Administration method - Oral administration	Gavage	1		
		Number of animals and sex distribution	Over 10 per group per male and female	1	15 per sex	
		Test type	Repeated dose toxicity study with a 2-generation reproductive and developmental toxicity study	1		
		Preliminary dose setting study	Performed	1	OECD 422 guideline-based design	
	Substance purity		Over 95% purity	5	potassium PFHxS (K+PFHxS) in deionized water, purity 99.98%	

Table S14. The RPF Reliability Evaluation for PFOS

Classification 1	Classification 2	Classification 3	Content	Score	Remarks	Reference
Data Confidence	Confidence in the determination of toxicity values		Else	0	There was no mention of it.	Bil et al. [27]
	Performing benchmark dose (BMD) modeling		The difference between BMD and BMDL is less than 2-fold	2.5	BMD(5) = 0.15 mg/kg/day; BMDL(5) = 0.098 mg/kg/day	
	Evaluating substances with read-across applied		-	0	Not applicable	
Study Confidence	Good laboratory practice (GLP) compliance status		Else	0	There was no mention of it.	Seacat et al. [48]
	Adequacy of toxicity testing	Test species	Animal (mouse, rat, etc.)	0.5	Sprague Dawley rats	
		Exposure duration	Subchronic (90 days to more than 1.5 years)	0.8	98 days	
		Administration method - Oral administration	Dietary: capsule or fed	0.2		
		Number of animals and sex distribution	3 – 9 per group per male and female	0.6	Male and Female, 5 per dose group	
		Test type	Repeated dose toxicity study	0.8		
		Preliminary dose setting study	Performed	1	Similar dose levels to those set in previous studies were used.	
	Substance purity		85-95 purity	3	PFOS potassium salt 86.9% pure	

References

1. ECHA. Per- and Polyfluoroalkyl Substances (PFAS). Available online: https://echa.europa.eu/hot-topics/perfluoroalkyl-chemicals-pfas (accessed on 5 May 2025).
2. Dirani, L.; Ayoub, G.M.; Malaeb, L.; Zayyat, R.M. A Review on the Occurrence of Per- and Polyfluoroalkyl Substances in the Aquatic Environment and Treatment Trends for Their Removal. *J. Environ. Chem. Eng.* **2024**, *12*, 113325. https://doi.org/10.1016/j.jece.2024.113325.
3. Perera, D.C.; Meegoda, J.N. PFAS: The Journey from Wonder Chemicals to Environmental Nightmares and the Search for Solutions. *Appl. Sci.* **2024**, *14*, 8611. https://doi.org/10.3390/app14198611.
4. Herzke, D.; Olsson, E.; Posner, S. Perfluoroalkyl and Polyfluoroalkyl Substances (PFASs) in Consumer Products in Norway—A Pilot Study. *Chemosphere* **2012**, *88*, 980–987. https://doi.org/10.1016/j.chemosphere.2012.03.035.
5. Newland, A.; Khyum, M.M.O.; Halamek, J.; Ramkumar, S. Perfluoroalkyl and Polyfluoroalkyl Substances (PFAS)—Fibrous Substrates. *Tappi J.* **2023**, *22*, 559–572. https://doi.org/10.32964/TJ22.9.559.
6. Mazumder, N.-U.-S.; Hossain, M.T.; Jahura, F.T.; Girase, A.; Hall, A.S.; Lu, J.; Ormond, R.B. Firefighters' Exposure to per-and Polyfluoroalkyl Substances (PFAS) as an Occupational Hazard: A Review. *Front. Mater.* **2023**, *10*, 1143411. https://doi.org/10.3389/fmats.2023.1143411.
7. Hashemi, S. Sanitation Sustainability Index: A Pilot Approach to Develop a Community-Based Indicator for Evaluating Sustainability of Sanitation Systems. *Sustainability* **2020**, *12*, 6937. https://doi.org/10.3390/su12176937.
8. United Nations. Sustainable Development Goal 6. Available online: https://sustainabledevelopment.un.org/sdg6 (accessed on 24 March 2020).
9. Von Behren, J.; Reynolds, P.; Bradley, P.M.; Gray, J.L.; Kolpin, D.W.; Romanok, K.M.; Smalling, K.L.; Carpenter, C.; Avila, W.; Ventura, A.; et al. Per- and Polyfluoroalkyl Substances (PFAS) in Drinking Water in Southeast Los Angeles: Industrial Legacy and Environmental Justice. *Sci. Total Environ.* **2024**, *953*, 176067. https://doi.org/10.1016/j.scitotenv.2024.176067.
10. Wee, S.Y.; Aris, A.Z. Environmental Impacts, Exposure Pathways, and Health Effects of PFOA and PFOS. *Ecotoxicol. Environ. Saf.* **2023**, *267*, 115663. https://doi.org/10.1016/j.ecoenv.2023.115663.
11. Bayode, A.A.; Emmanuel, S.S.; Akinyemi, A.O.; Ore, O.T.; Akpotu, S.O.; Koko, D.T.; Momodu, D.E.; López-Maldonado, E.A. Innovative Techniques for Combating a Common Enemy Forever Chemicals: A Comprehensive Approach to Mitigating per- and Polyfluoroalkyl Substances (PFAS) Contamination. *Environ. Res.* **2024**, *261*, 119719. https://doi.org/10.1016/j.envres.2024.119719.
12. Zahra, Z.; Song, M.; Habib, Z.; Ikram, S. Advances in Per- and Polyfluoroalkyl Substances (PFAS) Detection and Removal Techniques from Drinking Water, Their Limitations, and Future Outlooks. *Emerg. Contam.* **2025**, *11*, 100434. https://doi.org/10.1016/j.emcon.2024.100434.
13. Sharma, N.; Kumar, V.; Sugumar, V.; Umesh, M.; Sondhi, S.; Chakraborty, P.; Kaur, K.; Thomas, J.; Kamaraj, C.; Maitra, S.S. A Comprehensive Review on the Need for Integrated Strategies and Process Modifications for Per- and Polyfluoroalkyl Substances (PFAS) Removal: Current Insights and Future Prospects. *Case Stud. Chem. Environ. Eng.* **2024**, *9*, 100623. https://doi.org/10.1016/j.cscee.2024.100623.
14. Wickham, G.M.; Shriver, T.E. Emerging Contaminants, Coerced Ignorance and Environmental Health Concerns: The Case of Per- and Polyfluoroalkyl Substances (PFAS). *Sociol. Health Illn.* **2021**, *43*, 764–778. https://doi.org/10.1111/1467-9566.13253.
15. Fenton, S.E.; Ducatman, A.; Boobis, A.; DeWitt, J.C.; Lau, C.; Ng, C.; Smith, J.S.; Roberts, S.M. Per- and Polyfluoroalkyl Substance Toxicity and Human Health Review: Current State of Knowledge and Strategies for Informing Future Research. *Environ. Toxicol. Chem.* **2021**, *40*, 606–630. https://doi.org/10.1002/etc.4890.
16. Rudzanova, B.; Vlaanderen, J.; Kalina, J.; Piler, P.; Zvonar, M.; Klanova, J.; Blaha, L.; Adamovsky, O. Impact of PFAS Exposure on Prevalence of Immune-Mediated Diseases in Adults in the Czech Republic. *Environ. Res.* **2023**, *229*, 115969. https://doi.org/10.1016/j.envres.2023.115969.

17. US-EPA. Per- and Polyfluoroalkyl Substances (PFAS): Final PFAS National Primary Drinking Water Regulation. Available online: https://www.epa.gov/sdwa/and-polyfluoroalkyl-substances-pfas (accessed on 5 June 2025).
18. Hashemi, S.; Park, J.-H.; Yang, M.; Kim, J.; Oh, Y.; Pyo, H.; Yang, J. Long-Term Monitoring and Risk Assessment of N-Nitrosamines in the Finished Water of Drinking Water Treatment Plants in South Korea. *Environ. Sci. Pollut. Res.* **2022**, *29*, 3930–3943. https://doi.org/10.1007/s11356-021-15814-1.
19. Kim, M.; Jacob, M.F.; Muambo, K.E.; Sim, W.; Oh, J. Development of Simultaneous Analytical Methods of 37 PFAS in Drinking Water and Sediment Samples. *J. Environ. Anal. Health Toxicol.* **2022**, *25*, 58–70. https://doi.org/10.36278/jeaht.25.2.58.
20. Wee, S.Y.; Aris, A.Z. Revisiting the "Forever Chemicals", PFOA and PFOS Exposure in Drinking Water. *NPJ Clean Water* **2023**, *6*, 57. https://doi.org/10.1038/s41545-023-00274-6.
21. Yong, Z.Y.; Kim, K.Y.; Oh, J.-E. The Occurrence and Distributions of Per- and Polyfluoroalkyl Substances (PFAS) in Groundwater after a PFAS Leakage Incident in 2018. *Environ. Pollut.* **2021**, *268*, 115395. https://doi.org/10.1016/j.envpol.2020.115395.
22. Park, H.; Choo, G.; Kim, H.; Oh, J.-E. Evaluation of the Current Contamination Status of PFASs and OPFRs in South Korean Tap Water Associated with Its Origin. *Sci. Total Environ.* **2018**, *634*, 1505–1512. https://doi.org/10.1016/j.scitotenv.2018.04.068.
23. Schrenk, D.; Bignami, M.; Bodin, L.; Chipman, J.K.; del Mazo, J.; Grasl-Kraupp, B.; Hogstrand, C.; Hoogenboom, L.; Leblanc, J.; Nebbia, C.S.; et al. Risk to Human Health Related to the Presence of Perfluoroalkyl Substances in Food. *EFSA J.* **2020**, *18*, 6223. https://doi.org/10.2903/j.efsa.2020.6223.
24. US-EPA. *Human Health Toxicity Assessment for Perfluorooctanoic Acid (PFOA) and Related Salts. EPA Document No. 815-R-24-006*; U.S. Environmental Protection Agency: Washington, DC, USA, 2024.
25. US-EPA. *Human Health Toxicity Assessment for Perfluorooctane Sulfonic Acid (PFOS). EPA Document No. 815-R-24-007*; U.S. Environmental Protection Agency: Washington, DC, USA, 2024.
26. US-EPA. *Framework for Estimating Noncancer Health Risks Associated with Mixtures of Per- and Polyfluoroalkyl Substances (PFAS). EPA Document No. EPA-815-R-24-003*; U.S. Environmental Protection Agency: Washington, DC, USA, 2024.
27. Bil, W.; Zeilmaker, M.; Fragki, S.; Lijzen, J.; Verbruggen, E.; Bokkers, B. Risk Assessment of Per- and Polyfluoroalkyl Substance Mixtures: A Relative Potency Factor Approach. *Environ. Toxicol. Chem.* **2021**, *40*, 859–870. https://doi.org/10.1002/etc.4835.
28. Bil, W.; Ehrlich, V.; Chen, G.; Vandebriel, R.; Zeilmaker, M.; Luijten, M.; Uhl, M.; Marx-Stoelting, P.; Halldorsson, T.I.; Bokkers, B. Internal Relative Potency Factors Based on Immunotoxicity for the Risk Assessment of Mixtures of Per- and Polyfluoroalkyl Substances (PFAS) in Human Biomonitoring. *Environ. Int.* **2023**, *171*, 107727. https://doi.org/10.1016/j.envint.2022.107727.
29. Beck, N.B.; Becker, R.A.; Erraguntla, N.; Farland, W.H.; Grant, R.L.; Gray, G.; Kirman, C.; LaKind, J.S.; Jeffrey Lewis, R.; Nance, P.; et al. Approaches for Describing and Communicating Overall Uncertainty in Toxicity Characterizations: U.S. Environmental Protection Agency's Integrated Risk Information System (IRIS) as a Case Study. *Environ. Int.* **2016**, *89–90*, 110–128. https://doi.org/10.1016/j.envint.2015.12.031.
30. Ha, S.; Seidle, T.; Lim, K.-M. Act on the Registration and Evaluation of Chemicals (K-REACH) and Replacement, Reduction or Refinement Best Practices. *Environ. Health Toxicol.* **2016**, *31*, e2016026. https://doi.org/10.5620/eht.e2016026.
31. Hejgaard, C.K. Assessing Welfare of Rats Undergoing Gavaging with Varying Volumes. Measurements on Open Field Behaviour, Temperature, Plasma Corticosterone and Glucose. *Rev. Cienc.* **1999**, *23*, 16.
32. Diehl, K.; Hull, R.; Morton, D.; Pfister, R.; Rabemampianina, Y.; Smith, D.; Vidal, J.; Vorstenbosch, C. Van De A Good Practice Guide to the Administration of Substances and Removal of Blood, Including Routes and Volumes. *J. Appl. Toxicol.* **2001**, *21*, 15–23. https://doi.org/10.1002/jat.727.

33. Morton, D.B.; Jennings, M.; Buckwell, A.; Ewbank, R.; Godfrey, C.; Holgate, B.; Inglis, I.; James, R.; Page, C.; Sharman, I.; et al. Refining Procedures for the Administration of Substances. *Lab. Anim.* **2001**, *35*, 1–41. https://doi.org/10.1258/0023677011911345.
34. US-EPA. Guideline for Human Exposure Assessment. Available online: https://www.epa.gov/sites/production/files/2020-01/documents/guidelines_for_human_exposure_assessment_final2019.pdf (accessed on 14 October 2020).
35. Hashemi, S.; Shin, I.; Kim, S.-O.; Lee, W.-C.; Lee, S.-W.; Jeong, D.H.; Kim, M.; Kim, H.; Yang, J. Health Risk Assessment of Uranium Intake from Private Residential Drinking Groundwater Facilities Based on Geological Characteristics across the Republic of Korea. *Sci. Total Environ.* **2024**, *913*, 169252. https://doi.org/10.1016/j.scitotenv.2023.169252.
36. Korea Disease Control and Prevention Agency Korea National Health and Nutrition Examination Survey (KNHANES). Available online: https://knhanes.kdca.go.kr/knhanes/main.do (accessed on 28 April 2022).
37. Kweon, S.; Kim, Y.; Jang, M.-j.; Kim, Y.; Kim, K.; Choi, S.; Chun, C.; Khang, Y.-H.; Oh, K. Data Resource Profile: The Korea National Health and Nutrition Examination Survey (KNHANES). *Int. J. Epidemiol.* **2014**, *43*, 69–77. https://doi.org/10.1093/ije/dyt228.
38. Minnesota Department of Health. Air Toxicological Summary for: Perfluorohexanoic Acid (PFHxA). Available online: https://www.health.state.mn.us/communities/environment/risk/docs/guidance/air/pfhxa.pdf (accessed on 20 January 2025).
39. New Jersey Drinking Water Quality Institute. Health-Based Maximum Contaminant Level Support Document: Perfluorooctanoic Acid (PFOA). Available online: https://dep.nj.gov/wp-content/uploads/watersupply/bwse/dwqi-board/mcl-recs-and-reports/recs-for-mcl/pfoa/pfoa-appendixa.pdf (accessed on 20 January 2025).
40. New Jersey Drinking Water Quality Institute. Health-Based Maximum Contaminant Level Support Document: Perfluorononanoic Acid (PFNA). Available online: https://dep.nj.gov/wp-content/uploads/watersupply/bwse/dwqi-board/mcl-recs-and-reports/recs-for-mcl/pfna/a.pdf (accessed on 20 January 2025).
41. Texas Commission on Environmental Quality. Per- and Poly-Fluoroalkyl Substances (PFAS). Available online: https://www.tceq.texas.gov/downloads/toxicology/pfc/pfcs.pdf (accessed on 20 January 2025).
42. Minnesota Department of Health. Air Toxicological Summary for: Perfluorohexanesulfonic Acid (PFHxS). Available online: https://www.health.state.mn.us/communities/environment/risk/docs/guidance/air/pfhxs.pdf (accessed on 20 January 2025).
43. Minnesota Department of Health. Air Toxicological Summary for: Perfluorooctane Sulfonic Acid. Available online: https://www.health.state.mn.us/communities/environment/risk/docs/guidance/air/pfos.pdf (accessed on 20 January 2025).
44. Loveless, S.E.; Slezak, B.; Serex, T.; Lewis, J.; Mukerji, P.; O'Connor, J.C.; Donner, E.M.; Frame, S.R.; Korzeniowski, S.H.; Buck, R.C. Toxicological Evaluation of Sodium Perfluorohexanoate. *Toxicology* **2009**, *264*, 32–44. https://doi.org/10.1016/j.tox.2009.07.011.
45. Perkins, R.G.; Butenhoff, J.L.; Kennedy, G.L.; Palazzolo, M.J. 13-Week Dietary Toxicity Study of Ammonium Perfluorooctanoate (APFO) in Male Rats. *Drug Chem. Toxicol.* **2004**, *27*, 361–378. https://doi.org/10.1081/DCT-200039773.
46. Mertens, J.J.W.M.; Sved, D.W.; Marit, G.B.; Myers, N.R.; Stetson, P.L.; Murphy, S.R.; Schmit, B.; Shinohara, M.; Farr, C.H. Subchronic Toxicity of S-111-S-WB in Sprague Dawley Rats. *Int. J. Toxicol.* **2010**, *29*, 358–371. https://doi.org/10.1177/1091581810370372.
47. Butenhoff, J.L.; Chang, S.-C.; Ehresman, D.J.; York, R.G. Evaluation of Potential Reproductive and Developmental Toxicity of Potassium Perfluorohexanesulfonate in Sprague Dawley Rats. *Reprod. Toxicol.* **2009**, *27*, 331–341. https://doi.org/10.1016/j.reprotox.2009.01.004.

48. Seacat, A.M.; Thomford, P.J.; Hansen, K.J.; Clemen, L.A.; Eldridge, S.R.; Elcombe, C.R.; Butenhoff, J.L. Sub-Chronic Dietary Toxicity of Potassium Perfluorooctanesulfonate in Rats. *Toxicology* **2003**, *183*, 117–131. https://doi.org/10.1016/S0300-483X(02)00511-5.
49. Teymoorian, T.; Munoz, G.; Sauvé, S. PFAS Contamination in Tap Water: Target and Suspect Screening of Zwitterionic, Cationic, and Anionic Species across Canada and Beyond. *Environ. Int.* **2025**, *195*, 109250. https://doi.org/10.1016/j.envint.2025.109250.
50. Hong, S.; Kim, O.-J.; Jung, S.K.; Jeon, H.L.; Kim, S.; Kil, J. The Exposure Status of Environmental Chemicals in South Korea: The Korean National Environmental Health Survey 2018–2020. *Toxics* **2024**, *12*, 829. https://doi.org/10.3390/toxics12110829.
51. Choi, G.-H.; Lee, D.-Y.; Bruce-Vanderpuije, P.; Song, A.-R.; Lee, H.-S.; Park, S.-W.; Lee, J.-H.; Megson, D.; Kim, J.-H. Environmental and Dietary Exposure of Perfluorooctanoic Acid and Perfluorooctanesulfonic Acid in the Nakdong River, Korea. *Environ. Geochem. Health* **2021**, *43*, 347–360. https://doi.org/10.1007/s10653-020-00721-0.
52. Zhou, Y.; Chang, Y.; Zhang, D.; Li, W. Per- and Polyfluoroalkyl Substances in Potential Drinking Water Sources Globally: Distributions, Monitoring Trends, and Risk Assessment. *Water* **2025**, *17*, 3280. https://doi.org/10.3390/w17223280.
53. Munoz, G.; Liu, M.; Vo Duy, S.; Liu, J.; Sauvé, S. Target and Nontarget Screening of PFAS in Drinking Water for a Large-Scale Survey of Urban and Rural Communities in Québec, Canada. *Water Res.* **2023**, *233*, 119750. https://doi.org/10.1016/j.watres.2023.119750.
54. Hua, M.; McCauley, K.; Brew, D.; Heywood, J.; Siracusa, J.; Stevens, M.; Paustenbach, D. United States Environmental Protection Agency's Perfluorooctanoic Acid, Perfluorooctane Sulfonic Acid, and Related Per- and Polyfluoroalkyl Substances 2024 Drinking Water Maximum Contaminant Level: Part 1—Analysis of Public Comments. *Crit. Rev. Toxicol.* **2025**, *55*, 321–367. https://doi.org/10.1080/10408444.2024.2415893.
